# Local Adaptation Is Highest in Populations With Stable Long‐Term Growth

**DOI:** 10.1111/ele.70071

**Published:** 2025-02-18

**Authors:** Lauren N. Carley, Monica A. Geber, William F. Morris, Vincent M. Eckhart, David A. Moeller

**Affiliations:** ^1^ Department of Plant and Microbial Biology University of Minnesota Twin Cities St. Paul Minnesota USA; ^2^ Department of Ecology and Evolution The University of Chicago Chicago Illinois USA; ^3^ Department of Ecology and Evolutionary Biology Cornell University Ithaca New York USA; ^4^ Department of Biology Duke University Durham North Carolina USA; ^5^ Department of Biology Grinnell College Grinnell Iowa USA

**Keywords:** eco‐evolutionary dynamics, local adaptation, population dynamics

## Abstract

Theory suggests that the drivers of demographic variation and local adaptation are shared and may feedback on one other. Despite some evidence for these links in controlled settings, the relationship between local adaptation and demography remains largely unexplored in natural conditions. Using 10 years of demographic data and two reciprocal transplant experiments, we tested predictions about the relationship between the magnitude of local adaptation and demographic variation (population growth rates and their elasticities to vital rates) across 10 populations of a well‐studied annual plant. In both years, we found a strong unimodal relationship between mean home‐away local adaptation and stochastic population growth rates. Other predicted links were either weakly or not supported by our data. Our results suggest that declining and rapidly growing populations exhibit reduced local adaptation, potentially due to maladaptation and relaxed selection, respectively.

## Graphical abstract

Theory suggests that the drivers of demographic variation and local adaptation are shared and may feedback on one other. Using 10 years of demographic data and two reciprocal transplant experiments, we tested predictions about the relationship between the magnitude of local adaptation and demographic variation across 10 populations of a well‐studied annual plant. In both years, we found a strong unimodal relationship between mean home‐away local adaptation and stochastic population growth rates, suggesting that declining and rapidly growing populations exhibit reduced local adaptation.

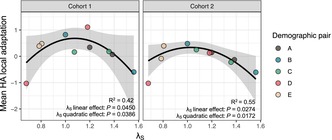

## 1 Introduction

An enduring goal in evolutionary biology is to understand the prevalence, magnitude and spatial scale of local adaptation (Briscoe Runquist et al. 2020; Clausen, Keck, and Hiesey 1940; Turesson 1922). Numerous reciprocal transplant experiments have documented local adaptation (Hereford 2009; Hoeksema and Forde 2008; Leimu and Fischer 2008), quantified as the fitness of a population at its home site relative to its fitness when transplanted to an away site (HA = ‘home‐away’), or the fitness of the local population at home relative to the fitness of a foreign population transplanted to that site (LF = ‘local‐foreign’) (Kawecki and Ebert 2004). It is well accepted that environmental variation shapes patterns of natural selection and that the magnitude of local adaptation is sometimes correlated with the environmental dissimilarity or geographic distance between populations or transplant sites (e.g., Hereford 2009; Bontrager and Angert 2019; Anderson and Wadgymar 2020; Gorton et al. 2022). Concurrently, population ecologists have explored how spatiotemporal environmental variation shapes population dynamics across species' ranges (Doak and Morris 2010; Eckhart et al. 2011; Ehrlén and Morris 2015; Schurr et al. 2012). Pursuit of these questions is increasingly motivated by the need to predict species' responses to global change.

Although typically assessed separately, the underlying drivers of demographic variation and local adaptation may be linked in at least two ways. First, a population's growth rate (λ), and whether it is declining (λ < 1), increasing (λ > 1) or stable (λ ≈ 1), may reflect and influence the process of adaptation. Environmental change can simultaneously cause population decline and result in maladaptation because phenotypic optima have shifted relative to the long‐term average. By contrast, environmental amelioration can cause rapid population growth and relaxed selection, which, in turn, can result in maladaptation and/or low local adaptation (Brady et al. 2019; Mukai et al. 1972; Shabalina, Yampolsky, and Kondrashov 1997). Second, demographic elasticities are related to selection gradients on life history components (i.e., vital rates) because they quantify the relative contribution of underlying vital rates to a population's intrinsic rate of increase, *r* (= ln λ), a measure of a population's mean fitness (de Kroon, van Groenendael, and Ehrlén 2000; Lande 1982; van Tienderen 2000). Similar patterns of vital rate elasticities between populations, reflecting similar patterns of life history selection, may therefore result in similar patterns of fitness and local adaptation across the landscape.

These relationships suggest two hypotheses that can be tested using classical approaches in evolutionary and population ecology: (1) rapidly‐declining or rapidly‐increasing populations should display lower average levels of local adaptation than stable populations and (2) dis/similarity in selection on life history between populations, as reflected in patterns of demographic elasticities, should be correlated with dis/similarity in patterns of local adaptation. To our knowledge, neither of these hypotheses has yet been explicitly tested.

Relationships between demography and natural selection are complex because demographic and adaptive processes can reciprocally affect each other (Hendry 2017; Pimentel 1968; Schoener 2011). Recent work has shown that evolutionary change influences population dynamics (Ives et al. 2020; Pelletier et al. 2017) and that ecological change drives rapid evolution (Benning, Faulkner, and Moeller 2023; Palkovacs, Mandeville, and Post 2014). However, most empirical work to date has leveraged short‐term and small‐scale perturbations of demographic or environmental conditions to test for these links (Hendry 2019), and it is unclear whether these eco‐evolutionary experiments approximate the dynamics of wild populations in complex environments. Longer‐term and larger‐scale experiments jointly quantifying population dynamics and local adaptation are necessary to assess the importance of eco‐evolutionary relationships in natural systems (Hendry 2019).

In this study, we integrated population ecological and evolutionary approaches to test for links between demography and local adaptation in a well‐studied annual plant, *Clarkia xantiana* A. Gray ssp. *xantiana* (Onagraceae; hereafter *C. xantiana* ). This species is an excellent study system because we have generated long‐term datasets on demographic and environmental variation across much of its geographic range, showing that stochastic population growth rates decline towards the species' eastern range limit in parallel with precipitation and temperature (Eckhart et al. 2011). Population genetic differentiation occurs over fine spatial scales (kilometres) and mirrors geography, with strong evidence of isolation‐by‐distance (Moeller, Geber, and Tiffin 2011; Pettengill, Briscoe Runquist, and Moeller 2016; Sianta, Moeller, and Brandvain 2024). Past experiments have also documented local adaptation of populations (Geber and Eckhart 2005; Benning and Moeller 2019), and fine‐scale genetic differentiation in morphological and phenological traits (Eckhart, Geber, and McGuire 2004; Gould et al. 2014) and in life history (Eckhart et al. 2011; Siegmund et al. 2023). In this study, we monitored 10 *C. xantiana* populations over 10 years to estimate population growth rates and their elasticities to underlying vital rates. We used field reciprocal transplant experiments, replicated in 2 years with contrasting abiotic conditions, to quantify fitness and local adaptation. We then leveraged complementary data on population dynamics and environment to answer two questions stemming from the two hypotheses regarding eco‐evolutionary links between demography and local adaptation:

(Q1) Are long‐term population growth rates quantitatively related to population‐mean levels of local adaptation?
(Q2) Do populations with similar elasticities display similar fitness, and less evidence of local adaptation, when transplanted into each other's home environments?

Recognising that different abiotic and biotic environments can favour similar life histories (i.e., similar patterns of life history selection/demographic elasticities), but that adaptation to these environments might be achieved through different traits or trait optima, we asked a third question:

(Q3) What are the relative contributions of demographic similarity, environmental similarity, and geographic proximity between populations in explaining patterns of local adaptation in reciprocal transplants?

## 2 Materials and Methods

### 2.1 Focal Populations

**FIGURE 1:**
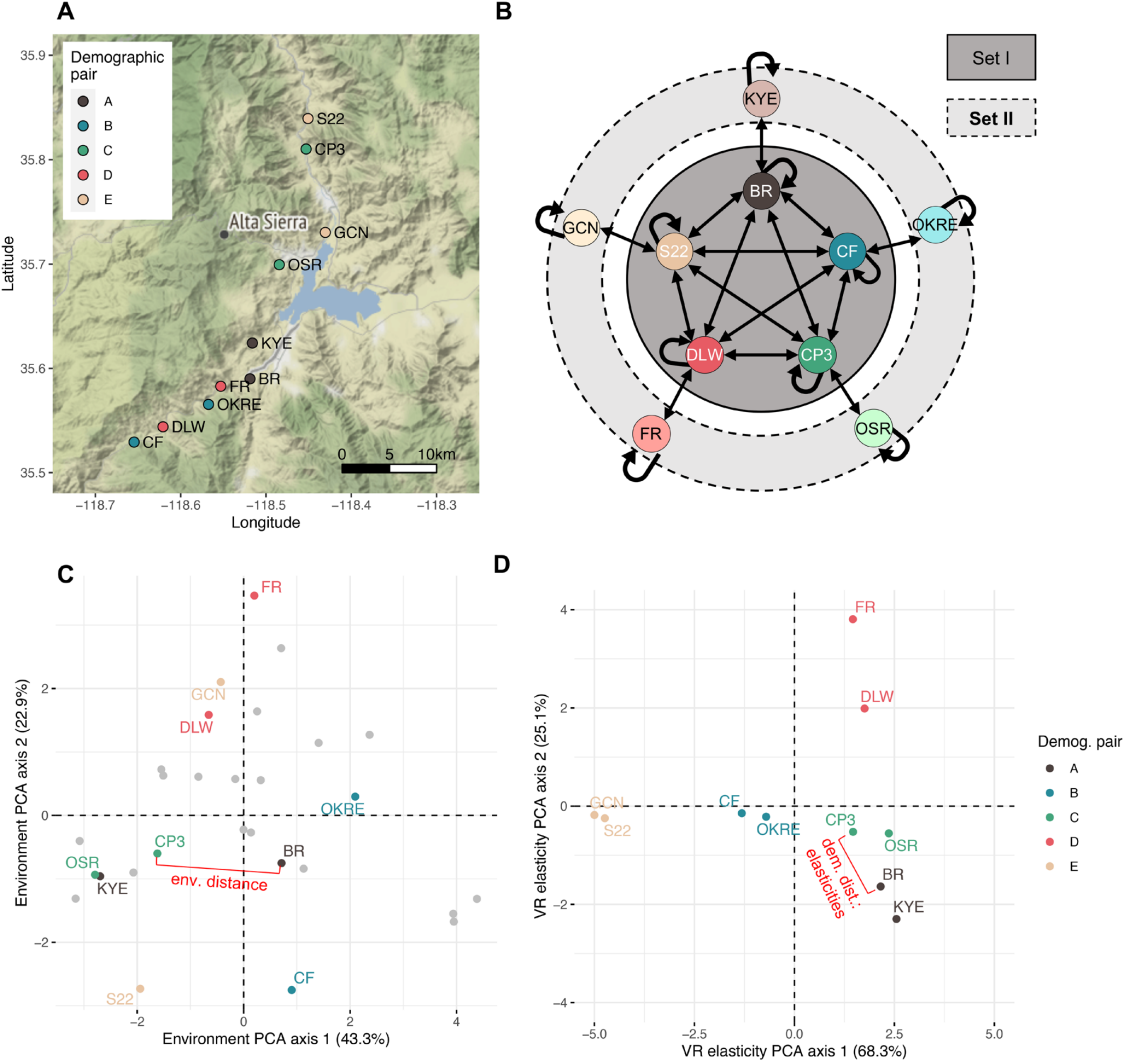
Overview of the *Clarkia xantiana* demographic reciprocal transplant experiment. (A) Focal populations in geographic space near Lake Isabella in Kern County, CA, USA. (B) A graphical model for the partially‐factorial field transplant design. Populations in the inner, darker circle comprise Set I, and were reciprocally transplanted into each other's sites. Populations in the outer, lighter circle comprise Set II, and were only reciprocally transplanted with their demographic pair from Set I. Arrows indicate transplant directions. (C) Focal populations in environmental space, as defined in a principal components analysis of temperature, precipitation, and solar irradiance across the ten focal sites (coloured points) and ten additional nearby sites across the species range (grey points). (D) Focal populations in demographic space, measured using a principal components analysis of the elasticity of each population's growth rate (λ) to twelve underlying vital rates (Appendices S1 and S2; Figure S4). Across all figure panes, populations depicted with the same point colour are demographic pairs. In panes (C, D), the red bracket depicts the continuous the estimation of the pairwise distance of each metric between one example pair of populations, Borel Road (BR) and Camp 3 (CP3).

In this study, we selected 10 focal *C. xantiana* populations that represent different combinations of long‐term population growth rate, patterns of vital rate elasticities, environments and geographic locations (Figure 1). The 10 populations are sources of seeds in the reciprocal transplant experiment and the populations' home sites are the locations of transplants.

#### 2.1.1 Demographic Variation

We used field census data from 2005 to 2016 and in situ germination experiments to estimate deterministic (λ) and stochastic (λ_S_) population growth rates for each population using the three‐stage matrix population model described in Eckhart et al. (2011) (Figure S1; see Appendix S1 for details of demographic modelling; range of λ: 0.88–1.82; range of λ_S_: 0.68–1.56; correlation between λ and λ_S_ = 0.93).

We used the elasticities of λ to 12 underlying vital rates as measures of selection on the mean vital rates of each population (Appendix S1). Because there is no established method for quantifying demographic (dis)similarity between pairs of populations, we compared three approaches, all involving a principal component analysis of vital rate elasticities or matrix element elasticities to first characterise variation in demographic space (see Appendices S1 and S2 for details). We then computed a dissimilarity metric as the Euclidean distance between pairs of populations in this demographic space. Because the three approaches yielded very similar distance values (pairwise correlations between distance metrics: *r =* 0.86–0.99; Figures S2 and S3), we used the distance in the demographic space defined by the first three axes of a principal component analysis of 12 vital rate elasticities (Figure 1D; Figure S4; *stats* ‘dist’; R Core Team 2020), which explained > 95% of variation in vital rate elasticities (Table S1).

Our 10 focal populations included two sets of five populations (Figure 1B). Five populations in ‘Set I’ are demographically dissimilar, i.e., distant in demographic space (Figure 1D). Each population in Set II is demographically similar to one Set I population, i.e., close to a Set I population in demographic space (Figure 1D).

#### 2.1.2 Environmental and Geographic Variation

To characterise environmental differences between site pairs, we used long‐term climate data from a network of weather stations deployed within and beyond the study area, supplemented by publicly available temperature and precipitation records, from 2005 to 2021 (Appendix S3). Briefly, using linear models (for temperature) and spatial interpolation (for precipitation), we estimated means and standard deviations of winter (November to January) and spring (February to June) daily temperature and of accumulated precipitation at each study sites. We estimated solar radiation at each site on the winter solstice and spring equinox in ArcGIS. We focused on these environmental data because they capture variation in temperature, moisture and light/exposure during the time of year that spans the majority of the species' aboveground life history. We characterised the major axes of environmental variation across sites with a principal component analysis (*stats* ‘prcomp’; R Core Team 2020) of the 10 variables (Figure S5). We estimated the environmental distance between pairs of populations as the pairwise Manhattan distance in the PCA space defined by the first five PCA axes, which accounted for > 95% of environmental variation (‘dist’ function in the *stats* package in R; R Core Team 2020; Table S2).

We calculated pairwise geographic distances between populations/transplant site pairs using the geodesic method (*geodist* in R; Padgham and Sumner 2021). Geographic distance is a proxy both for genetic distance (Pettengill, Briscoe Runquist, and Moeller 2016; Sianta, Moeller, and Brandvain 2024) and for unmeasured features of the environment.

Demographic, geographic and environmental distances between populations pairs were not significantly correlated (pairwise correlation coefficients | *r* | = 0.07–0.30; *p* = 0.11–0.71; Figure S3).

### 2.2 Field Transplant Experiments

To estimate the magnitude of local adaptation across populations, we performed a two‐tiered reciprocal transplant experiment with Set I and Set II populations (Figure 1B). Seeds from each Set I population were transplanted into the home site, the sites of the four other Set I populations, and the site of the demographically similar population from Set II. Seeds from each Set II population were transplanted into the home site and the site of the population's demographic pair in Set I. This two‐tiered approach allowed us to estimate local adaptation in a broad range of populations without requiring a fully factorial 10 × 10 transplant design, which exceeded logistical and space constraints.

We repeated the transplant experiment in 2 years (Cohort 1: 2016 and Cohort 2: 2019). We collected seeds from 1 to 5 fruits from 50 to 100 maternal plants, haphazardly selected at least 1 m apart in each population in June of each cohort year. We pooled seeds across maternal plants within source populations, and randomly selected from these mixed pools when planting.

For each cohort, we transplanted ~30,000 seeds across the 10 transplant sites in November, with each source population represented 760–792 times per site, distributed across 96 blocks at Set I sites and 33 blocks at Set II sites (Table S3). We planted one seed per 3.8 cm^2^ cell of 49‐cell plastic grids, with each grid comprising one randomised, complete statistical block. Grids were affixed to the soil surface and filled with local soil from > 30 cm below the surface; soil from this depth contains few *C. xantiana* seeds and minimises contamination from the natural population.

We tracked the fitness of each transplanted seed by recording its germination, survival and reproduction from February through June. For Cohort 1, we estimated reproductive success by counting seeds in all fruits on each surviving plant. For Cohort 2, we estimated seed number from fruit mass, because seed number and fruit mass are tightly correlated (Figures S6 and S7; Table S4). Full details of planting and data collection are provided in Appendix S4.

### 2.3 Data Analysis

#### 2.3.1 Fitness in Reciprocal Transplants

We quantified the absolute lifetime fitness (*W*_*i,j,k*_) of each transplanted seed, *k*, from each source population *i* at transplant site *j*. Lifetime fitness, defined as the number of seeds produced per planted seed, was estimated using *aster* models, which account for contingencies of fitness components across life history (Geyer, Wagenius, and Shaw 2007; Shaw et al. 2008). Because of the partially factorial nature of the experimental design (Figure 1B), we fit 10 *aster* models (one per transplant site) for each cohort (Appendix S4).

We estimated the mean fitness of population *i* transplanted to site *j* (*W*_*i,j*_) as the average *aster*‐derived absolute fitness off all *i* seeds at site *j*. Because seeds can remain dormant but still germinate and reproduce in subsequent years, our models estimate the lifetime fitness of seeds that germinate in the first year following seed dispersal. Population mean fitness, *W*_*i,j*_, is equivalent to the upper left element of the demographic projection matrix (matrix element *a*_*11*_; Figure S1; Appendix S1).

#### 2.3.2 Estimating Local Adaptation

We compared patterns of lifetime fitness across all seed source and transplant site combinations to estimate the magnitude of local adaptation using both HA and LF fitness contrasts. HA contrasts compare the fitness of a population at home to its fitness in ‘away’ transplant sites, while LF contrasts compare the fitness of a local population at home to the fitness of non‐local (‘foreign’) populations in the same site. Positive values of either metric reflect local adaptation because they indicate that a population has higher fitness at home than at other sites (HA local adaptation) and/or has higher fitness at home than do foreign populations at that site (LF local adaptation). Negative values reflect maladaptation. The magnitudes of these values reflect the strength of local (mal)adaptation.

Detailed methods describing HA contrasts are provided below. Because LF contrasts yielded similar but weaker patterns of local adaptation, we present full details on parallel LF analyses in Appendix S5. For HA contrasts, we first relativised mean population fitness within sites for each population *i* grown in site *j*:

(1) $$\varphi_{i,j}=\frac{W_{i,j}}{{\bar{W}}_{∙j}}$$

where ${\bar{W}}_{∙j}$ is the average absolute fitness of all source populations planted at site *j*. Relativising a population's fitness within sites for HA contrasts controls for possible differences in the quality of sites that can affect the absolute fitness of all populations (Blanquart et al. 2013). To estimate HA_*i,j*_, we then subtracted population *i*'s relative fitness in the away site *j* (*j* ≠ *i*) from its relative fitness at home site *i*:

(2) $${\mathrm{HA}}_{i,j}=\varphi_{i,i}-\varphi_{i,j\neq i}$$

Hereafter, we use the phrase ‘pairwise fitness contrasts’ to mean HA_*i,j*_ (Equation 2) or LF_*i,j*_ (Appendix S5).

We then estimated the mean magnitude of local adaptation expressed by each population (Blanquart et al. 2013) as:

(3) $${\bar{\mathrm{HA}}}_{i}=\varphi_{i,i}-\frac{1}{S-1}\;\sum_{j\neq i}\varphi_{i,j}$$

where *S* is the number of sites each population was tested in. Unlike pairwise fitness contrasts (Equation 2), this metric describes a population's mean magnitude of local adaptation, informed by patterns of local adaptation expressed across all tested source‐site pairs.

#### 2.3.3 Q1: Population‐Mean Local Adaptation

To answer Q1, we fit a multiple regression modelling population‐mean local adaptation (Equation 3) in response to linear and quadratic effects of λ_S_, allowing for possible non‐linear relationships. We fit separate regressions for each transplant cohort. Because more information is available to estimate mean local adaptation in Set I than Set II populations, we used weighted regressions with a weight of 5 for Set I populations and 1 for Set II populations. We used ANOVA and permutation tests to assess the significance of the regressions (Appendix S6).

#### 2.3.4 Q2: Comparing Demographic Pairs

To answer Q2, we fit linear models examining the effect of demographic pair status of populations *i* and *j* on pairwise fitness contrasts (Equation 2), with separate models for each transplant cohort. In HA_*i,j*_ contrasts, ‘demographic pair status’ indicates whether the source population *i* is demographically similar to the population local to the away transplant site *j* (Figure 1). For LF_*i,j*_ contrasts, demographic pair status indicates whether the source population *i* is the demographic pair of the foreign population *j* grown at site *i*. We tested the effect of demographic pair status on pairwise fitness contrasts for each transplant cohort using ANOVA and permutation tests (Appendix S6), predicting that the magnitude of HA and LF contrasts will be lower when *i* and *j* are demographic pairs.

#### 2.3.5 Q3: Relative Contributions of Geographic, Environmental and Demographic Similarity to Local Adaptation

To answer Q3, we fit multiple linear regressions of pairwise fitness contrasts in response to standardised pairwise demographic, environmental and geographic distances, with separate regressions for each transplant cohort. We tested the effect of each distance metric on pairwise fitness contrasts using ANOVA and permutation tests (Appendix S6).

## 3 Results

### 3.1 Reciprocal Transplant Outcomes

**FIGURE 2:**
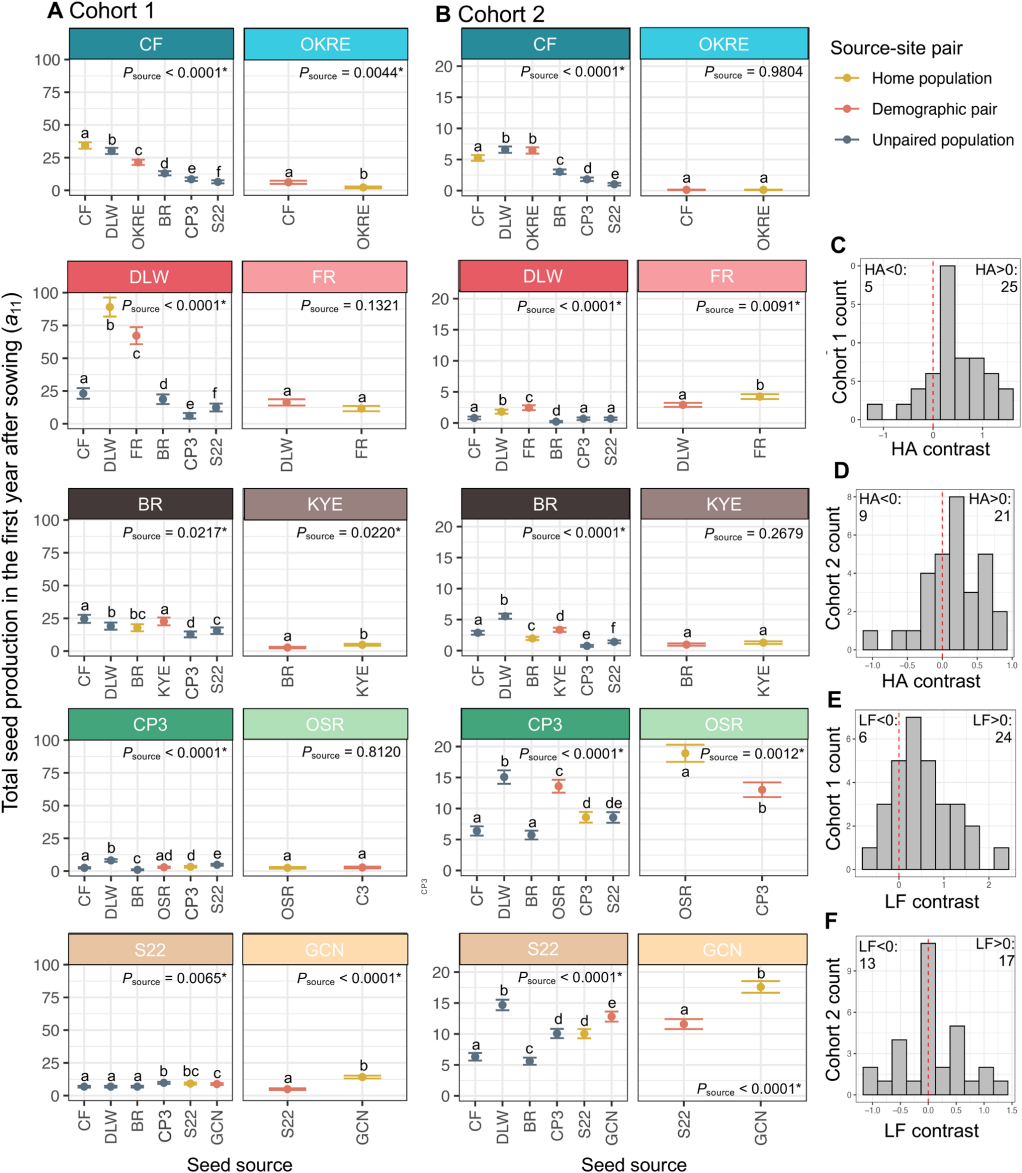
(A, B) Mean population absolute fitness (*W*_*i,j*_), as predicted in site‐specific *aster* models, in each source‐site transplant combination in (A) Cohort 1 and (B) Cohort 2 of the field transplant experiment. Note changes on y‐axis scale between cohorts. The left column of panels in (A) and (B) show the five Set I transplant sites, ordered by longitude from west (top) to east (bottom). The right column of panels shows the paired Set II sites. Text strips denoting each transplant site are colour‐coded to denote population pairs according to Figure 1. Mean fitness values and error bars (± 1 standard error) in each panel are colour‐coded to show the home population (yellow) and foreign demographically‐paired (orange) and demographically‐unpaired (blue) populations. *p* values in figure panels denote the significance of population seed source in determining lifetime fitness in a given transplant site, based on analysis of deviance comparing the goodness‐of‐fit of a model containing population seed source (*W*_*i,j,k*_ *= β*_*0*_ *+ β*_*1*_ *I*, where I designates 1 of 6 source populations at Set I transplant sites or 1 of 2 source populations at Set II transplant sites) vs. a model excluding the main effect of population seed source; full statistical details are provided in Table S5. An * appears next to *p* values < 0.05. Lower case letters approximate pairwise significant differences in absolute lifetime fitness across source populations within transplant sites; for Set I populations, source populations are considered to have significantly different fitness if the modelled mean falls outside of the range of other source populations' mean ± 1 SE. (Traditional post hoc pairwise contrasts are not compatible with *aster* models). For Set II populations, because only two source populations are compared within each transplant site, populations are considered to have significantly different fitness if the main effect of source population was significant in the *aster* models. (C–F) Histograms show variation in HA and LF pairwise fitness contrasts within each transplant cohort.

Our partially factorial reciprocal transplant experiment (Figure 1B) generated fitness estimates for 40 source‐site combinations, replicated across two cohorts that experienced different abiotic conditions. Within transplant sites, population source influenced absolute fitness in 16 out of 20 tested transplant sites, demonstrating genetic variation for fitness across populations (Figure 2; Table S5). Absolute fitness was substantially lower in Cohort 2, coinciding with a 60% reduction in total growing season precipitation relative to Cohort 1 (Figure S8). Despite this, absolute fitness exceeded replacement (*W* = 1) for nearly all source‐site combinations in both cohorts. The majority of pairwise fitness contrasts were positive (Figure 2C–F), whether using the HA metric Equation (2) or the LF metric. This suggests that the majority of *C. xantiana* populations in this study express some level of local adaptation, although the strength of local adaptation varies across populations.

### 3.2 Q1: Predictors of Population‐Mean Local Adaptation

**FIGURE 3:**
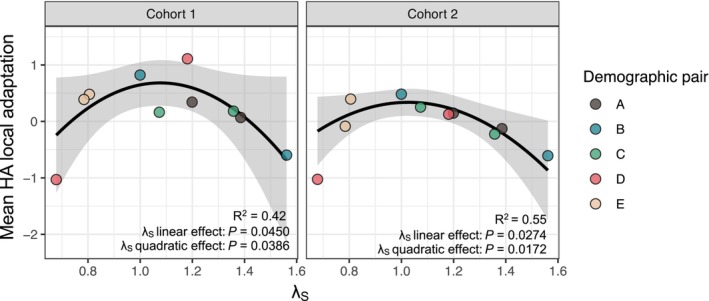
Population‐mean levels of HA adaptation were strongly correlated with λ_S_, the long‐term population growth rate, in both transplant cohorts. Colours of data points indicate population pairs as in Figure 1. Black and grey bands represent the fitted line and 95% prediction interval for a weighted regression accounting for differences in the amount of data informing estimates of population‐mean local adaptation for Set I vs. Set II populations. *p* values in each panel represent significance of linear and quadratic effects of λ_S_ in multiple regressions (${\bar{\mathrm{HA}}}_{i}$ = *β*_*0*_ *+ β*_*1*_ λ_S_(*i*) + *β*_*2*_ λ_S_^2^(*i*)), based on permutation tests to account for partial non‐independence of fitness contrasts (Appendix S6). Full statistical details are provided in Table S6.

The long‐term population growth rate (λ_S_) was nonlinearly correlated with population‐mean HA local adaptation: populations with λ_S_ ≈ 1 expressed the highest mean local adaptation in both cohorts (Figure 3; Table S6), and populations with λ_S_ either well below or well above 1 showed lower mean local adaptation. Population growth rates did not predict population mean LF local adaptation, although the relationship was similarly shaped for Cohort 1 (Table S7; Figure S9).

### 3.3 Q2: Fitness Contrasts Between Demographic Pairs

**FIGURE 4:**
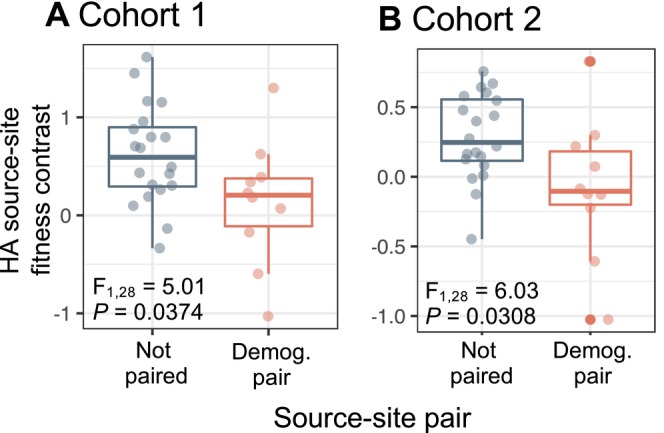
Pairwise home‐away fitness contrasts (*HA*_*i,j*_) across source‐site combinations of populations grown at home (*i*) vs. away sites (*j*). When population *i* is transplanted to an away site, *j*, belonging to its demographic pair, datapoints and boxplots are shown in orange; when not, datapoints and boxplots are shown in blue. Values greater than 0 indicate that home‐site fitness exceeds away‐site fitness for a given source‐site combination. Conversely, values equal to or less than 0 indicate that a population's away‐site fitness is equal to or greater than its home‐site fitness, respectively. For each cohort, the effect of demographic pair status (PS_*i,j*_) on pairwise fitness contrasts was analysed in an ANOVA model: *HA*_*i,j*_ = *β*_*0*_ *+ β*_*1*_ PS_*i,j*_. *p* values in each panel represent the significance of pair status based on permutation tests to account for partial non‐independence of fitness contrasts (Appendix S6). Full statistical details are provided in Table S8.

Compared to fitness at home, populations suffered less of a fitness cost (i.e., showed reduced HA local adaptation) when grown at sites of their demographic pair than at sites of demographically‐dissimilar populations (Figure 4; Table S8). Patterns were not statistically significant for LF contrasts (Figure S10).

### 3.4 Q3: Relative Contributions of Demography, Environment and Geography to Local Adaptation

**FIGURE 5:**
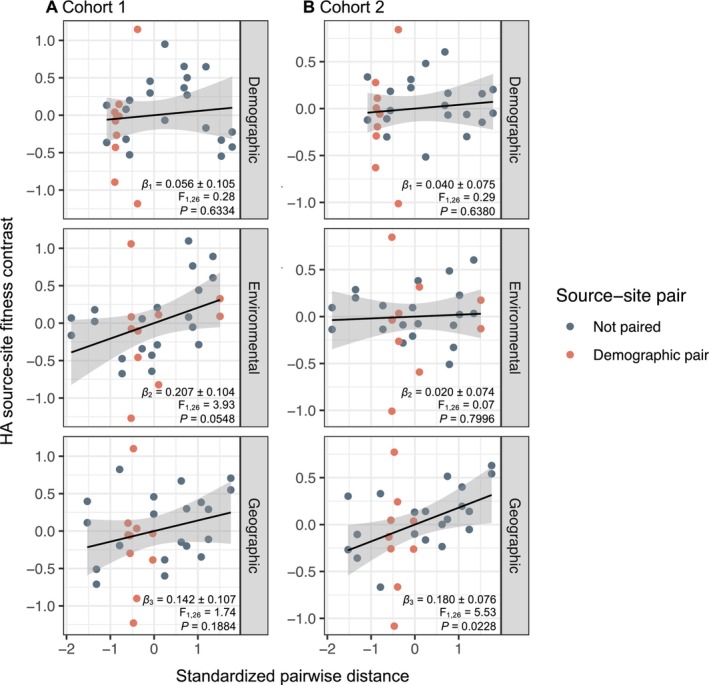
Effects of standardised demographic distance (dd_*i,j*_), environmental distance (ed_*i,j*_), and geographic distance (gd_*i,j*_) on pairwise HA fitness contrasts (Equation 2) among source‐site combinations in (A) Cohort 1 and (B) Cohort 2 of the field transplant experiment. Each column of panes reflects a multiple regression model fitting HA fitness contrasts in response to all three putative drivers of local adaptation (*HA*_*i,j*_ = *β*_*0*_ *+ β*_*1*_ dd_*i,j*_ + *β*_*2*_ ed_*i,j*_ + *β*_*3*_ gd_*i,j*_). Within each panel, data points and fitted lines reflect partial correlations between each driver and HA fitness contrasts. We visualised partial correlations by plotting the residual values of a model fitting HA contrasts in response to both other independent variables vs. the residual values taken from a model fitting the predictor of interest (named in each row) against both other independent variables. In the models shown here, demographic distance is estimated as the pairwise distance in vital rate elasticity space (Figure 1D). *β* estimates in each panel represent the partial regression coefficients (± 1 standard error) from the full multiple regression model fitting pairwise HA contrasts in response to all three pairwise distances. *F* statistics are derived from the empirical model for each multiple regression. *p* values represent the significance of each distance metric based on permutation tests to account for partial non‐independence of fitness contrasts (Appendix S6). Full statistical details are provided in Table S9.

Quantitative pairwise demographic distances did not predict pairwise HA fitness contrasts in either cohort (Figure 5; Table S9). In Cohort 1, environmental distance was marginally correlated with HA contrasts: populations had marginally reduced fitness costs (i.e., showed reduced HA local adaptation) when grown at away sites that were environmentally similar to their home site (Figure 5A, middle). In Cohort 2, geographic distance was positively correlated with HA contrasts, such that the fitness reduction at an away site relative to home increased with geographic distance (Figure 5B, bottom). None of the three pairwise distance metrics predicted LF pairwise fitness contrasts (Table S10).

## 4 Discussion

Previous work has identified theoretical and empirical links between demography and local adaptation (see Introduction; Gazave et al. 2013; Gravel 2016; Klopfstein, Currat, and Excoffier 2006; de Kroon, van Groenendael, and Ehrlén 2000; Lohmueller 2014; Metcalf and Pavard 2007; Peischl et al. 2013; Peischl et al. 2018; van Tienderen 2000). However, such eco‐evolutionary relationships are typically studied over short time scales and in controlled environments. Here, we used several approaches to test for quantitative links between demography and local adaptation over 10 years. Our results show that source populations had similar fitness in home and away sites when the home and away populations were demographic pairs. By contrast, continuous estimates of demographic similarity failed to predict pairwise fitness contrasts, which were better predicted by geographic distance. Nevertheless, population‐mean local adaptation was non‐linearly correlated with long‐term population stability, with the highest magnitude of local adaptation observed in populations where λ_S_ ≈ 1. These results suggest that some eco‐evolutionary relationships persist in natural conditions and across larger spatiotemporal scales than are typically assessed. However, our results depart considerably from studies in simplified environments and suggest that realistic environments modify the strength and predictability of eco‐evolutionary links.

### 4.1 Nonlinear Relationships Between Demography and Local Adaptation

Several mechanisms may plausibly cause limited mean local adaptation in populations with low growth rates. First, selection may not yield local adaptation in small populations where genetic drift erodes genetic variation for fitness (reviewed in Barton and Turelli 2004; Willi, VanBuskirk, and Hoffman 2006; but see Goodnight 1987; Willis and Orr 1993; Wood, Yates, and Fraser 2016). Second, long‐term population growth rates may be low because the mean annual growth rate is low and/or because there is high temporal variation in annual growth rates (Lewontin and Cohen 1969; Tuljapurkar and Orzack 1980). Populations with consistently low growth rates may be maladapted to their current environments, e.g. because gene flow links populations experiencing divergent selection (Bachmann et al. 2020) or because response to selection cannot track shifting optima under environmental change (Anderson and Wadgymar 2020; Gorton et al. 2022). Populations with high interannual variation in growth rates may experience strong temporal fluctuations in natural selection, which can erode genetic variation over time (Hedrick 1976), reducing adaptive capacity. Third, populations with greater adaptive phenotypic plasticity may have lower mean HA local adaptation because individual fitness is similar across environments. However, it is unclear whether and how population‐level variation in plasticity might relate to population growth rates.

That populations with high long‐term growth rates have reduced local adaptation is a more surprising finding, but is consistent with theoretical predictions. Rare genetic variants tend to increase in frequency during population growth (Marth et al. 2004; Klopfstein, Currat, and Excoffier 2006; François et al. 2008), including deleterious mutations (Gazave et al. 2013; Lohmueller 2014). For example, population growth during range expansion and/or following population bottlenecks often results in elevated genetic load (Keinan and Clark 2012; Peischl et al. 2013; Wang et al. 2018). Rapid population growth may also be driven by environmental conditions that are similarly favourable for all genotypes in a population, resulting in relaxed selection and reduced local adaptation or maladaptation (Brady et al. 2019; Mukai et al. 1972; Shabalina, Yampolsky, and Kondrashov 1997).

In *C. xantiana* , *N*_*e*_ is quite large even in smaller or geographically marginal populations, likely because populations are primarily outcrossing and have seed banks (Moeller, Geber, and Tiffin 2011). Thus, it is unlikely that small *N*_*e*_ and/or limited genetic variation for fitness has hampered adaptation. However, shifting environmental drivers of selection may explain maladaptation in *C. xantiana* populations with low growth rates. This study occurred during the most severe megadrought in the recorded history of the southwestern United States, which has been exacerbated by climate change (Williams et al. 2020; Williams, Cook, and Smerdon 2022). Drought‐induced selection has elicited adaptive responses in some *C. xantiana* populations, but others have failed to track changing phenotypic optima through time (Benning, Faulkner, and Moeller 2023). Thus, temporal changes in contemporary environmental conditions may enhance local adaptation in some populations while hindering it in others, consistent with variation in the extent of local adaptation detected across populations in this study. Some *C. xantiana* populations show signatures of rapid population expansion in recent evolutionary history (Pettengill, Briscoe Runquist, and Moeller 2016); thus, increases in the frequency of rare and potentially deleterious mutations may also reduce local adaptation in populations with high λ_S_. Relaxed selection in environments where population growth has been rapid is also a possibility, although future work is needed to test this explanation.

Finally, while the quantitative relationship between λ_S_ and local adaptation is striking, the direction of causality in this relationship remains unclear. Above, we describe ways in which population (in)stability may constrain or facilitate local adaptation. However, adaptation may also drive population dynamics; for example, locally adapted populations may have stable or increasing growth rates, while maladaptation may cause populations to decline. These explanations reasonably describe *C. xantiana* populations with low and stable growth rates but fail to explain why some populations with lower magnitude of local adaptation have high growth rates.

### 4.2 Inference From Different Analytical Approaches

In addition to identifying a relationship between λ_S_ and population‐mean local adaptation, analyses using categorical assignments of demographic similarity upheld the hypothesis that populations express similar fitness when grown in the environments of demographically similar populations. However, on a continuous scale, demographic distance was uncorrelated with source‐site fitness contrasts. How do population‐level relationships between demography and local adaptation arise when demographic similarity is an inconsistent predictor of fitness in reciprocal transplant experiments?

First, it is possible that similarity in past selection on life history components across populations may contribute relatively little to contemporary patterns of fitness. *Clarkia xantiana* populations exhibit fine‐scale differentiation in traits such as morphology and phenology (Eckhart, Geber, and McGuire 2004; Gould et al. 2014). If selection favours similar life histories across populations but the traits that achieve those life histories differ across populations (e.g., due to spatial variation in the environmental drivers of local adaptation), life history variation may not directly relate to local adaptation. Second, if population dynamics directly influence local adaptation (e.g., population growth or fluctuations drive the process of adaptation), elasticities may fail to capture the demographic variation that is relevant to local adaptation. Third, it is possible that shared selection on life history does contribute to variation in local adaptation, but that our elasticity analyses failed to describe this well. Because the structure of the demographic model is the same across all focal populations (Appendix S1), overall patterns of elasticities may be qualitatively similar among populations (Morris and Doak 2005). Fourth, if among‐population differences in vital rates are driven by plastic responses to environmental conditions, the elasticity of λ to vital rates may indirectly describe environmental effects rather than past natural selection.

Finally, we acknowledge that the reciprocal transplant design from which we estimate local adaptation captures individuals that germinate in the first year following seed dispersal (demographic matrix element *a*_*11*_; Figure S1B). Because *C. xantiana* is an annual species, this accurately captures lifetime fitness of seeds that germinate without dormancy. However, this could underestimate lifetime fitness for individuals and populations that remain viable in the seed bank for one or more years following dispersal. Our focal populations vary considerably in seed bank dynamics, and variation in delayed germination contributed to both quantitative estimation of demographic distance and qualitative assignments of populations to demographic pairs (Figures S2 and S4 and Table S1). For populations with greater dormancy, underestimation of the absolute fitness values used to calculate HA and LF contrasts may have limited our power to detect relationships between demographic similarity and local adaptation. Indeed, relationships that we did detect were stronger in Cohort 2 (Figures 4 and 5), which had higher germination rates in the reciprocal transplant (Table S11). Thus, relationships between life history variation and local adaptation may be more difficult to measure in populations that have, or environments that induce, longer seed dormancy. In these cases, multi‐year studies may be necessary to accurately measure total fitness even in annual species. In this study, logistical constraints limited our ability to reliably track the fates transplanted seeds for more than 1 year following sowing, so we were unable to measure the survival and reproduction of seeds that failed to germinate in the first year following sowing but remained viable.

Our findings also show strong relationships between HA local adaptation and demography, but weaker or inconsistent relationships between LF adaptation and demography. We used both metrics in all analyses because there is unresolved debate about which metrics are most informative or biologically relevant (Kawecki and Ebert 2004; Blanquart et al. 2013; Bachmann and Van Buskirk 2020). While the two metrics were correlated (Figure S11), HA contrasts were more consistent across transplant years (Figure S12) and overall had stronger relationships to demographic variation. This does not necessarily suggest that the HA metric is more informative writ large, but these findings raise the possibility that local adaptation estimated by comparing the fitness of one population in a variety of sites may more closely reflect the effects of demographic processes on evolution than does local adaptation estimated by comparing the fitness of multiple populations within a site.

### 4.3 Non‐Demographic Effects on Local Adaptation

Recent work suggests that the level of local adaptation detected in transplant experiments is associated with the degree of similarity in historical climate variables between the seed source and transplant site (Anderson and Wadgymar 2020; Bontrager and Angert 2019; DeMarche, Angert, and Kay 2020; Gorton et al. 2022; Angert, Bontrager, and Ågren 2020; Bontrager et al. 2021; Halbritter et al. 2018; Hereford 2009; Leimu and Fischer 2008). In addition, these environmental distance effects are often stronger predictors of local adaptation than geography alone. Our data also show that HA fitness contrasts were marginally related to environmental distance between source populations and transplant sites; however, this relationship was weak and only detected in Cohort 1 (which had a substantially wetter winter than other years in recent history; Figure S8).

In Cohort 2, only geographic distance between source populations and growth sites was a significant predictor of pairwise HA fitness contrasts. This relationship could reflect higher levels of gene flow and/or greater shared ancestry between geographically proximate populations. Populations of *C. xantiana* are indeed linked by gene flow and exhibit isolation by distance (Moeller, Geber, and Tiffin 2011; Pettengill, Briscoe Runquist, and Moeller 2016; Sianta, Moeller, and Brandvain 2024). We were unable to use pairwise genetic distances in our analyses because earlier work did not include all focal populations from this study; we instead use geographic distance as the best available proxy. High gene flow could prevent or slow adaptation by repeatedly introducing alleles that confer maladaptation, particularly in a heterogeneous landscape. However, we only detected a relationship between pairwise fitness contrasts and geographic distance in Cohort 2, whereas patterns in Cohort 1 were marginally correlated with environmental distance. Thus, it is also possible that unmeasured environmental variation that covaries with geography (e.g., biotic interactions; Benning et al. 2019) may drive the relationship between local adaptation and geographic distance.

### 4.4 Conclusions and Implications

Our work provides novel evidence for a quantitative link between population stability and mean local adaptation across a natural landscape and over multiple generations. However, we found minimal evidence for a relationship between the degree of source‐site local adaptation and demographic similarity based on elasticities. Our results suggest that modifying factors such as gene flow may complicate tight relationships between life history variation and adaptation. Because eco‐evolutionary feedbacks can modulate the pace of range expansion, our results have implications for responses to climate change. Our finding that populations with high long‐term growth rates showed reduced rather than enhanced mean local adaptation implies that range expansion may decelerate due to such eco‐evolutionary feedbacks, which contrasts with most predictions from theory and empirical studies in simplified environments (Miller et al. 2020). Additional long‐term studies in wild populations would be helpful to further evaluate the prevalence of eco‐evolutionary feedbacks and the spatial and temporal scales over which they are important.

## Author Contributions

**Lauren N. Carley:** data curation, formal analysis, investigation, visualisation, writing – original draft, writing – review and editing. **Monica A. Geber:** conceptualisation, formal analysis, funding acquisition, investigation, methodology, project administration, resources, supervision, validation, writing – original draft, writing – review and editing. **William F. Morris:** formal analysis, funding acquisition, software, writing – reviews and editing. **Vincent M. Eckhart:** conceptualisation, data curation, funding acquisition, investigation, writing – reviews and editing. **David A. Moeller:** conceptualisation, formal analysis, funding acquisition, investigation, methodology, project administration, resources, supervision, validation, writing – original draft, writing – review and editing.

## Conflicts of Interest

The authors declare no conflicts of interest.

## Supporting information


Appendices S1–S6

Figures S1–S13

Tables S1–S11

## Data Availability

Data and code supporting this manuscript are archived in the Dryad Digital Repository (doi: 10.5061/dryad.f1vhhmh24).
