# Supplementary material for: Local Adaptation Is Highest in Populations With Stable Long‐Term Growth
Source: Ecol Lett. 2025 Feb 18;28(2):e70071. doi: 10.1111/ele.70071 (PMC11834371; doi:10.1111/ele.70071)
Supplement: Supplementary file 1 — Appendices S1–S6 [file ELE-28-0-s003.docx]

# Appendices

**“Local adaptation is highest in populations with stable long-term growth”**

L.N. Carley, M. A. Geber, W. F. Morris, V. Eckhart, and D. A. Moeller*

*correspondence: [moeller@umn.edu](mailto:moeller@umn.edu)

Contents:

Appendix S1: Demographic modeling methods……………………………………………………….2

Appendix S2: Pairwise demographic distance calculations………………………………………….4

Appendix S3: Environmental data analyses…………………………………………………………...5

Appendix S4: Detailed transplant experiment methods………………………………………………7

Appendix S5: Local-foreign contrasts...…………………………………………………………...…...8

Appendix S6: Permutation tests……………………………………………………………………….11

## Appendix S1: Demographic modeling methods

### Vital rate measurements

We censused the 10 focal populations twice annually from 2005-2016 as described in Eckhart et al. (2011). Briefly, we counted the number of new seedlings in 30 permanent quadrats in each site in February of each year. We then revisited the same quadrats in June of each year to determine the number of individuals who survived to reproduction (*σ*), and the number of fruits produced per surviving plant (*F*), and sampled fruit number on plants from supplemental transects. We estimated the number of seeds produced per fruit (φ) by collecting fruits from plant on these each June and counting seeds in the laboratory, accounting for differences in seed production for fruits that did and did not experience frugivory. The supplemental transects were located away from focal quadrats so as to not reduce seed dispersal into the permanent census plots. We used seed bag burial experiments replicated across three years (2005/6, 2006/7, and 2007/8) to estimate germination of seed in the first, second and third winter following seed production (*g*_1_*, g*_2_*,* and *g*_3_) and dormancy (1–*g*_1_, 1–*g*_2_, 1–*g*_3_). We also estimated seed survival from fall to winter (*s*_1_, *s*_3_, *s*_5_), and winter to fall (*s*_2_, *s*_4_, *s*_6_) in the year following production and over the next two years. Seed survival from production in July to October burial in the seed bag experiment (*s*_0_) was extrapolated from seed bag data, and the input of seeds into, and emergence of seedlings from, permanent plots (see Eckhart et al. 2011 for details).

### Estimation of λ and λ_S_

Using the vital rate data described above, we constructed a population projection matrix model (Caswell 2001) for each of the ten focal populations based on a life-cycle graph composed of three stages corresponding to seed age in October (**Supplementary Figure 1A**). Our three-stage matrix model accounts for the survival and reproduction of all individuals present aboveground as well as the recruitment of new aboveground individuals from seeds germinating from seed bank seeds in their first, second, or third or later year following dispersal. We used the vital rate estimates to construct a projection matrix for each population for each year of the demographic study (using means of belowground vital rates for years without seed bag data). We then estimated the deterministic population growth rate, λ, as the dominant eigenvalue of the mean projection matrix for each population (**Supplementary Figure 1B**), as described in Eckhart et al. (2011). Finally, we estimated the long-term stochastic population growth rate, λ_s_, by randomly choosing one matrix each year from among the set of annual matrices for each population, projecting the population forward one year at a time for 50,000 years (by which time the λ_s_ estimate had stabilized), and computing the geometric mean of the 50,000 annual growth rate estimates (Caswell 2001). Numerical simulation of λ_S_ using the 10 annual matrices preserves the variances of, and the covariances between, the annual vital rate estimates. The estimates λ and λ_s_ were strongly and linearly correlated across the 10 populations used in the transplant experiment (*r* = 0.93, *F*_1,8_ = 49.2, *P* < 0.0002; **Supplementary Figure 13**).

### Elasticity analyses

From our demographic models, we performed elasticity analyses to assess how changes in projection matrix elements and underlying vital rates affect changes in λ in each population.

Using standard procedures (Caswell 2001), we first calculated the elasticities, *e*_ij_, of λ to the projection matrix elements, *a*_ij_ (**Supplementary Figure 1B**). We used these values to calculate elasticities, *e*_j_, of λ to underlying vital rates, including seed survival (*s*_1_ - *s*_6_), seed germination (*g*_1_ - *g*_3_), and aboveground survival and reproduction (*σ*, *F*, *φ*) (**Supplementary Figure 1C**). While the matrix element elasticities, *e*_ij_, sum to 1, the vital rate elasticities, *e*_i_, do not.

Elasticities quantify the proportional change in λ elicited by an infinitesimal proportional change in a matrix element or vital rate holding all other elements or vital rates constant (de Kroon et al. 2000; van Tienderen 2000). Thus, *e*_ij_, the elasticity of λ to matrix element *a*_ij_, is the partial derivative of ln(λ) with respect to ln(*a*_ij_), or the partial regression slope of ln(λ) on ln(*a*_ij_), in the vicinity of the estimated ln(*a*_ij_), and is related to the standardized selection gradient on matrix element *a*_ij_ (van Tienderen 2000, p. 668: *e*_ij_ = *a*_ij_ × β_ij_, where β_ij_ is the selection gradient on *a*_ij_). In other words, *e*_ij_ is related to the strength of direct directional selection on *a*_ij_. Similar calculations with similar implications for selection on vital rates apply to elasticities of λ to vital rates, *e*_j_ (van Tienderen 2000).

Finally, we calculated the elasticity of λ to the four life-cycle loops that make up the life cycle of *C. xantiana* (**Supplementary Figure 1D**). A life cycle loop is a pathway that starts and ends at the same stage in an organism’s life cycle (van Groenendael et al. 1994; Wardle 1998; de Kroon et al. 2000). For example, in an annual plant’s life cycle in which seeds can germinate within a year of being produced (i.e., there is no dormancy) or remain dormant for one or more years before germinating, the first life cycle loop starts with a newly produced seed that germinates within the first year, proceeds through seedling survival to reproduction and fruiting, and ends with newly produced seed. In our projection matrices, this loop is described as *a*_11_, and is equivalent to the fitness measure estimated in the reciprocal transplant experiment, which incorporates the likelihood of seed germination (or not) and survival and fecundity in the first year following seed sowing. The other life cycle loops describe seeds that remain dormant in the seed bank for one, two, or three or more years, respectively, before germinating. Unlike individual life history components which may contribute to one or more matrix elements, or individual matrix elements that comprise only a portion of a life cycle pathway, life cycle loops describe complete life-history pathways. We calculated elasticities of loops according to previous methods (Wardle 1998, Guneralp 2007). As with elasticities of matrix elements, the elasticities of life-cycle loops sum to 1.

We used these vital rate and loop elasticity values to calculate demographic distance metrics reflecting (dis)similarity across populations in patterns of vital rate elasticities or loop elasticities, using several approaches (**Appendix S2**).

## Appendix S2: Pairwise demographic distance calculations

Unlike quantifying geographic or environmental distances/dissimilarities among populations (Bontrager & Angert 2019; Anderson & Wadgymar 2020; DeMarche et al. 2020; Gorton et al. 2022), to our knowledge there are no established methods to quantify demographic (dis)similarity among populations. We compared three metrics for describing demographic distances among our focal populations.

In Method A, we used elasticities of the 4 life cycle loops to describe how populations differ in the contributions of these loops to λ. We calculated pairwise demographic distances between populations in 4-dimensional elasticity space (1 dimension for the elasticity of each of the four life cycle loops; **Supplementary Figure 1D; Supplementary Figure 2A**), using the Manhattan method.

In Method B, we used the elasticities of 12 underlying vital rates to describe how populations differ in the contributions the vital rates to λ. We omitted the elasticity of the vital rate s0, seed survivorship from dispersal in July to the initiation of seed bag experiments, because s0 is derived from other vital rates (**Appendix S1**). We performed a principal component analysis of the 12 elasticities and calculated pairwise demographic distance between populations as the Euclidean distance in 3-dimensional PCA space defined by the first three PCA axes (**Figure 1D**). In Method B, our vital rate elasticity principal components analysis separated populations based on the importance of immediate vs. 1-year-delayed vs. 3-or-more-year-delayed vital rates in driving λ (**Supplementary Figure 4; Supplementary Table 1**).

In parallel with Method B, we also computed demographic distances between pairs of populations using stochastic elasticities that measure λ_s_’s elasticity to proportional changes in the 12 vital rates, accounting for inter-annual variation in the vital rates (Tuljapurkar et al. 2003; Haridas & Tuljapurkar 2005). Demographic distances based on stochastic elasticities were similar to those based on deterministic elasticities.

In Method C, we focused on elasticities of two vital rates, *g*_1_ and *F*, which contribute prominently to lifetime fitness as measured in the reciprocal transplant experiment (**Appendix S1**). Vital rates *σ* (survival from seedling to reproductive stage), and φ (seeds produced per fruit) also contribute to lifetime fitness, as measured in the reciprocal transplant experiment. However, because *F, σ*, and φ contribute to the same matrix element (**Supplementary Figure 1B-C**), and because vital rate elasticities are derived from the matrix element elasticities (see above), the elasticity values for *F, σ*, and φ are numerically identical. We therefore chose to contrast a single elasticity for these three vital rates with the elasticity of *g_1_* to describe how populations differ in λ’s elasticity to these facets of first year fitness. We calculated pairwise Euclidean distances between populations in 2-dimensional *g*_1_ vs. *F* space (**Supplementary Figure 2B**).

Demographic distances between pairs of populations estimated by these three methods were strongly positively correlated (*r* = 0.86-0.99). We therefore present results based on Method B metric in the main text.

## Appendix S3: Environmental data analyses

We quantified environmental variation across focal populations using three data types: long-term temperature records, long-term precipitation records, and solar radiation on select dates.

### Component data

#### Climate

We characterized variation in temperature and precipitation among the study populations as described in Eckhart et al. (2011) and Gould et al. (2014), expanding the temporal and spatial record. Briefly, beginning in 2005, we deployed environmental sensors (Onset Computer Corporation, Bourne, MA, USA) that logged shaded atmospheric temperature and tipping-bucket precipitation at 30 min intervals. We maintained sensors at up to 20 locations per year, including 4 locations beyond the eastern range edge and at the sites of 7 of the 10 populations reported on here (CF, FR, BR, KYE, OSR, GCN, and S22). Filtering raw data converted values to daily average temperatures and total daily precipitation, which could then be collected into several-month “seasons” corresponding to the *C. xantiana* life cycle (see below). To increase the spatial density of sampling, we downloaded publicly available daily (or monthly) records from weather stations maintained by state and federal agencies (California Data Exchange Center). Accidents (e.g., lightning, theft, vandalism, livestock damage, instrument failure) left 8-20 of our own stations operating at any given time, supplemented by 2-6 “public” stations.

To summarize climate for periods relevant to the *Clarkia xantiana* life cycle, we binned temperature and precipitation data into three seasons (“winter” = 1 November to 31 January; “spring” = 1 February to 30 June; and “summer” = 1 July to 31 October). We focused here on winter (when seed germination begins) and spring (from germination to flowering and seed maturation). Seeds disperse passively in summer, lying in the soil until winter rains begin.

We used ArcGIS software (Desktop or Pro, ESRI, Redlands, CA, USA) to estimate temperature and precipitation. To model temperature, we estimated for each weather station in the network its elevation, linear azimuth (absolute value of slope aspect deviating from true south), and its solar radiation on each solstice and on the vernal equinox. Predictive models were general linear models (run in Minitab, State College, PA) of station temperatures on elevation, linear azimuth, and winter-solstice solar radiation (for winter temperatures) or on elevation, linear azimuth, and spring equinox and summer solstice radiation (for spring temperatures), including year as a random variable. In this way, we estimated seasonal temperatures for every one-hectare pixel in the 57 km tall x 48.7 km wide study area for every year and season, 2005-2021, given pixels’ elevation, linear azimuths, and solar radiation values. In every case, elevation accounted for the majority of variance in seasonal temperature. Extracting temperature values from population points was the final step. To model seasonal precipitation in each year, we interpolated between points by inverse distance weighting, which, for this application, has the advantage of being precisely accurate for the sites of population’s stations in that year. For any focal populations geographically larger than one pixel, we used the modeled value from the pixel at the approximate center of the population. We used modeled temperature and precipitation values from our network of sensors for each focal population, including those that had dataloggers present, since even sites with direct observations sometimes had missing data due to instrument damage and equipment failures.

For each of the populations in the present study we calculated long-term means and standard deviations of temperature and precipitation during winter and spring seasons from the 2005-2021 estimates. This process yielded eight climate variables (two seasons [winter and spring] × two variables [mean temperature and cumulative precipitation] × two summary statistics [mean and SD]) for use in broader environmental analysis.

#### Solar radiation

To characterize variation in exposure and sunlight across sites, we extracted solar radiation estimates from ArcGIS as described previously (Gould et al. 2014). As in our climate analyses, to incorporate variation most meaningful to the *C. xantiana* life cycle, we focused on solar radiation estimates from the winter and spring seasons (estimated at the winter solstice and spring equinox, respectively). We included these data in environmental analyses because irradiance varies across this species’ range in ways that are often decoupled with climate (pers. obs.), and may influence photosynthesis, survival, growth, and reproduction.

### Principal component analysis and pairwise environmental distances

To describe the major axes of environmental variation across sites, we performed a principal components analysis on the combined climate data (long-term mean and variability in temperature and precipitation during winter and spring seasons) and solar radiation (irradiance at winter solstice and spring equinox), after centering and scaling all data (**Figure 1B**). This PCA primarily separated populations based on mean and long-term variability in winter precipitation and spring temperature (**Supplementary Figure 5; Supplementary Table 2**).

We used the Manhattan method for estimating environmental distances because >3 PCA axes were required to explain >95% of environmental variation, vs. 3 or fewer axes being sufficient for capturing geographic and demographic distance; the Manhattan method may perform better than the Euclidean method in higher-dimensionality datasets (Aggarwal et al. 2001). Standardized pairwise environmental distances estimated using the Manhattan vs. Euclidean methods were very highly correlated (*r* = 0.90), and outcomes of downstream analyses using the two methods were nearly identical.

Pairwise demographic, geographic, and environmental distance metrics were weakly correlated (| *r* | = 0.07-0.30; **Supplementary Figure 3**).

## Appendix S4: Detailed transplant experiment methods

### Planting

For Set I transplant sites, each grid contained 8 seeds from each of 6 source populations (the local Set I population, its Set II demographic pair, and the four other Set I populations). For Set II transplant sites, each grid contained 24 seeds from each of two source populations (the local Set II population and its Set I demographic pair). Within grids, seed planting positions were randomized. Full details on precise sample sizes in each site and year are provided in **Supplementary Table 3.**

We minimized incursion of non-transplant seeds into experimental grids by weeding wild *C. xantiana* individuals within a 1-m perimeter of each transplant grid prior to planting. We also filled grids with local soil collected from ≥ 30 cm below the surface to avoid including local seeds from the seed bank in experimental grids. We included one empty cell per grid as a control to check for contamination with local seeds. We excluded from analyses individual grid cells from which >1 seedling emerged, and whole grids if the control cell had a germinant or >2 cells in the grid contained >1 germinant. 27 out of 623 control cells in Cohort 1 (4.17%) and 27 out of 634 control cells in Cohort 2 (4.26%) had a germinant in them.

### Field data collection

For the first cohort, seeds were transplanted in November of 2016. We measured germination, early life survival, adult survival and flowering, and fruit production during repeated censuses in February, March, May, and June of 2017. At the end of the growing season, we collected all fruits produced by transplanted individuals that survived to reproduction.

For the second cohort, seeds were transplanted in November of 2019. Subsequent fieldwork was limited due to the COVID-19 pandemic; we measured germination in February 2020 and late-season survival in June 2020. In June we also collected all fruits of surviving individuals. While not directly observed, we inferred flowering in 2020 from fruiting data; all individuals that produced fruits were counted as having flowered, and individuals that died after flowering but prior to fruit production were noted during the June census.

In both cohorts, germination was recorded beginning in February and re-assessed at each subsequent census. In other words, our data captures germination occurring at “typical” times for this winter annual species (January-February) as well as “late” germination (March-May). We are able to detect germination of individuals that do not survive to establishment because dead germinants remain visible in transplant grids for several months (pers. obs.). Thus, our estimation of early life-history fitness components in the reciprocal transplants captures much of the available information about germination and seedling survival within the first year of sowing.

### Seed number estimation

In the first transplant cohort, we directly counted the total number of seeds produced by each fruiting individual to quantify reproductive output. Because fruit mass and seed number were highly correlated in Cohort 1, in Cohort 2 we estimated total reproductive output by predicting seed number from fruit mass.

When collecting fruits in Cohort 2, we noticed that a subset of fruits displayed evidence of herbivore damage to fruits (frugivory) which may influence the relationship between fruit mass and seed set. As such, we identified each fruit categorically as undamaged or damaged during collection. We then separately weighed all undamaged and damaged fruits from each fruiting individual.

We selected a stratified random sample of 30 individuals spanning the full observed range of fruit mass in each site-source combination and counted their seeds. We counted seed number and measured fruit mass separately for undamaged and damaged fruits, and regressed seed number on fruit mass for the two fruit types using linear models (**Supplementary Figure 6**). We used the estimated model coefficients (**Supplementary Table 4**) to predict seed number from undamaged and from damaged fruits, based on their measured masses, for all other fruiting individuals in that source-site combination. We summed the seeds from the two fruit types and rounded the sum to the nearest whole number to estimate total reproductive output for each fruiting plant.

For the subset of plants in year 2 for which we both weighed fruits and counted seeds, we tested the precision of these models by comparing predicted and observed seed numbers using a linear regression. Predicted and observed values were tightly correlated (**Supplementary Figure 7**).

### ASTER models

For each site-specific model in Cohort 1, we employed the following model structure:

| Root | → | Germination | → | March survival | → | May survival | → | Flowering | → | Fruiting | → | Seeds |
| --- | --- | --- | --- | --- | --- | --- | --- | --- | --- | --- | --- | --- |
|  |  | Bernoulli (0,1) |  | Bernoulli  (0,1) |  | Bernoulli (0,1) |  | Bernoulli (0,1) |  | Bernoulli (0,1) |  | Poisson |

In Cohort 2, because fewer life-history transitions were directly observed, we eliminated the May survival node from the model, as follows:

| Root | → | Germination | → | March survival | → | Flowering | → | Fruiting | → | Seeds |
| --- | --- | --- | --- | --- | --- | --- | --- | --- | --- | --- |
|  |  | Bernoulli (0,1) |  | Bernoulli  (0,1) |  | Bernoulli (0,1) |  | Bernoulli (0,1) |  | Poisson |

Finally, at one site in Cohort 2 (Golf Course North, i.e. GCN), all individuals that flowered fruited, causing those two life-history transitions to be aliased. For this site, we used a further reduced model structure, eliminating the flowering node:

| Root | → | Germination | → | March survival | → | Fruiting | → | Seeds |
| --- | --- | --- | --- | --- | --- | --- | --- | --- |
|  |  | Bernoulli (0,1) |  | Bernoulli  (0,1) |  | Bernoulli (0,1) |  | Poisson |

We quantified the absolute lifetime fitness of each transplanted seed, *k*, for each source population *i* at transplant site *j*, *W_i,j,k_*, as the number of seeds produced per planted seed, using site-specific *aster* models. We tested the effect of source population at each site on individual absolute lifetime fitness by using an analysis of deviance to compare the goodness-of-fit of a model containing population seed source (*W_i,j,k_ = β_0_ + β_1_ I,* where *I* designates 1 of 6 source populations at Set I transplant sites or 1 of 2 source populations at Set II transplant sites*)* vs. a model excluding the main effect of population seed source. We used the fitted aster models, including source population effects, to estimate the mean and standard error of lifetime fitness in each source-site combination.

## Appendix S5: Local-foreign contrasts

### Methods

#### Estimating local adaptation

In addition to home-away fitness contrasts described in the main text, we estimated the magnitude of local adaptation using local-foreign (LF) fitness contrasts. For LF fitness contrasts, we relativized fitness of population *i* grown in site *j* by the population’s average fitness across all tested transplant sites $\bar{W}_{i\cdot}$:

$$\varphi_{i,j}^{'}= \frac{W_{i,j}}{\bar{W}_{i \cdot}} \left( Eq. S1 \right)$$

Relativization of a population’s fitness in this manner controls for possible differences in the quality of populations that can affect a population’s absolute fitness at all tested sites (Blanquart et al. 2013). (Note that $\varphi_{i,j}$ presented in the Methods for calculating HA*_i,j_* and ${\varphi'}_{i,j}$ presented here for calculating LF*_i,j_* are different quantities.) We then estimated the magnitude of local adaptation using LF pairwise contrasts, by subtracting the relative fitness of a foreign population (*j* ≠ *i*) from the relative fitness of the local population at site *i*:

$${LF}_{i,j}= {\varphi'}_{i,i}-{\varphi^{'}}_{j\neq i,i} (Eq. S2)$$

#### Population-mean local adaptation

In addition to pairwise fitness contrasts, we estimated the mean magnitude of LF local adaptation for each population (Blanquart et al. 2013):

$$\bar{LF}_{i}= {\varphi'}_{i,i}-\frac{1}{P-1}\sum_{j\neq i} {\varphi^{'}}_{j,i} (Eq. S3)$$

where *P* is the number of populations tested within each site. Unlike pairwise fitness contrasts (Eq. S2), $\bar{LF}_{i}$ estimates the fitness of the home population in site *i* minus the mean fitness of all foreign populations tested at site *i*.

### Results

#### Q1: Predictors of population-mean local adaptation

Long-term population stability was not correlated with population-mean LF in either transplant cohort (**Supplementary Table 7**), although in Cohort 1 the pattern was qualitatively similar to the hump-shaped relationship detected with population-mean HA local adaptation (**Supplementary Figure 9**).

#### Q2: Fitness contrasts between demographic pairs

Pairwise LF contrasts showed qualitatively similar patterns to HA contrasts across source-site pairs in Cohort 1, but the effect of demographic pair status was only marginally significant (**Supplementary Table 8B; Supplementary Figure 10**). In Cohort 2, LF contrasts for demographic pairs were no smaller than LF contrasts for demographically dissimilar populations.

#### Q3: Relative contributions of demography, environment, and geography to local adaptation

In multiple regressions examining the effects of demographic vs. geographic and environmental similarity on local adaptation, no drivers significantly predicted LF source-site contrasts in either cohort (**Supplementary Table 10**).

## Appendix S6: Permutation tests

HA and LF pairwise fitness involving the same home/local population and site (e.g., HA*_i,j_*/LF*_i,j_* and HA*_i,k_*/LF*_i,k_*) are not independent of each other because they are calculated in reference to the same measurement of home ($\varphi_{i,i})$ or local fitness (${\varphi'}_{i,i})$. To account for this non-independence in analyses of the drivers of pairwise fitness differences, we performed permutation tests where we randomly shuffled the absolute first-year fitness (*W*) for each source-site combination without replacement within each cohort. We then repeated the calculations of site- and population-wise relative fitness (Eq. 1, Eq. S1), pairwise fitness contrasts (Eq. 2, Eq. S2), and mean local adaptation (Eq. 3, Eq. S3) as described above, using the randomized values.

We used the resulting randomized estimates to repeat downstream analyses for the three questions. We repeated the randomization process 5,000 times and extracted the *F* statistic for each effect of interest in each downstream model. We then compared the empirical *F* statistic for each effect of interest to the distribution of *F* values generated in the 5,000 randomizations, and assessed significance based on whether the observed *F* exceeded the cutoff for the upper 5% tail of the permuted distribution (i.e., α = 0.05).

**References appearing in the supporting information**

Aggarwal, C. C., Hinneburg, A., Keim, D. A. (2001). On the surprising behavior of distance metrics in high dimensional space. *In*: Van den Bussche, J., Vianu, V. (eds) Database Theory — ICDT 2001. ICDT 2001. Lecture Notes in Computer Science, vol 1973. Springer, Berlin, Heidelberg. <https://doi.org/10.1007/3-540-44503-X_27>

Caswell, H. (2001) *Matrix Population Models: Construction, Analysis, and Interpretation.* United Kingdom: Sinauer Associates.

Guneralp, B. (2007) An improved formal approach to demographic loop analysis. *Ecology* 88: 2124-2131.

van Groenendael, J., de Kroon, H., Kalisz, S. & Tuljapurkar, S. (1994) Loop analysis: evaluating life history pathways in population projection matrices. Ecology 75: 2410-2415.

Wardle, G. M. (1998) A graph theory approach to demographic loop analysis. *Ecology* 79: 2539-2549.
