# Supplementary material for: Local Adaptation Is Highest in Populations With Stable Long‐Term Growth
Source: Ecol Lett. 2025 Feb 18;28(2):e70071. doi: 10.1111/ele.70071 (PMC11834371; doi:10.1111/ele.70071)
Supplement: Supplementary file 2 — Figures S1–S13 [file ELE-28-0-s002.docx]

## Supplementary figures

**Supplementary Figure 1.** A description of the demographic model used to estimate *C. xantiana* population dynamics. **A:** Life cycle graph. **B**: Demographic matrix structure. **C:** Matrix transitions as defined by underlying vital rates: *σ*: survival of seedlings from germination to fruiting; *F*: fruit number per plant; *φ*: seed number per fruit; *s_0_*: seed survival in soil from production in June to the start of seed bank experiments in October, *s_1_* survival from the first October to the first germination opportunity in February, *g_1_*: germination of first year seed in February; *s_2_* survival of ungerminated seed from February to the second October; *s_3_*: survival of second year seed to second germination opportunity in February of second year, *g_2_*: germination of second year seed; *s_4_*: survival of non-germinated seed to third October; *s_5_*: survival of seed to the third germination opportunity in February of third year; *g_3_*: germination of third year seed; s6: survival of ungerminated seed from February to the fourth October. **D**: Life cycle loops as defined by underlying matrix elements. In this annual species, population structure arises from seed bank dynamics, with discrete stages represented as circles in panel **A** and matrix elements in panel **B**.

**Supplementary Figure 2.** Comparisons of demographic distance estimated using method A (**A**) and method C (**B**). Method B is visualized in **Figure 1D**. Across both panels, populations shown in the same colors were assigned as demographic pairs for the reciprocal transplant experiment. All tested methods placed demographic pairs near to one another in demographic space (**Appendix S2**).

**Supplementary Figure 3.** Plot of correlations among population pairwise distance metrics. In each box, ellipses represent correlation strengths, with tighter, darker ellipses showing stronger correlations and wider, lighter ellipses showing weaker correlations. Positive relationships are shown in blue, and negative relationships are shown in red. Above the diagonal, overlaid orange text shows pairwise correlation coefficients (*r*). Below the diagonal, overlaid black text shows *P*-values representing the significance of correlations.

**Supplementary Figure 4.** Loading of underlying vital rate elasticities onto axes 1 and 2 of the demographic PCA defined by 12 vital rates. Vital rates are defined in **Appendix S1** and **Supplementary Figure 1**.

**Supplementary Figure 5.** Loading of underlying variables onto axes 1 and 2 of the environmental PCA. Across all vectors, abbreviations are as follows: SD = standard deviation, T = temperature, P = precipitation, Rad = solar radiation; subscripts are as follows: W = winter, S = spring.

**Supplementary Figure 6.** Linear regressions of seed number on fruit mass from Cohort 2 of the reciprocal transplant experiment. Separate regression models were fit for fruits without (**a**) and with (**b**) herbivore damage in each site (facets) and source population (color and pattern) combination.

**Supplementary Figure 7.** Correlation between predicted and observed seed number among individuals for whom seeds were counted in Cohort 2 of the reciprocal transplant experiment. Symbol shapes indicate source populations, and symbol colors indicate the transplant site. The red dashed line denotes a 1:1 relationship between predicted and observed values.

**Supplementary Figure 8.** Variation in temperature (top) and precipitation (bottom) in the winter (left) and spring (right) seasons throughout our environmental record (2005-2021). Thin gray lines show the yearly trends in seasonal climatic variation across populations, plotted against longitude (west to east, from the range center toward the eastern range edge). The thick lines show the fitted relationships regressing each variable on longitude across all years of data (black line), in Cohort 1 of the transplant (blue line), and Cohort 2 of the transplant (orange line). Notably, transplants in Cohort 1 experienced a particularly wet winter that reversed the typical shallow decline in precipitation toward the range edge.

**Supplementary Figure 9.** Relationships between population-mean LF local adaptation and long-term population growth rates. Colors of data points indicate population pairs as in **Figure 1**. Black and gray bands represent the fitted line and 95% prediction interval for a weighted regression accounting for differences in the amount of data informing estimates of population-mean local adaptation for Set I vs. Set II populations (see **Methods**). *P* values in each panel represent significance of linear and quadratic effects of λ_S_ in multiple regressions $\bar{(LF}_{i}$ = *β_0_ + β_1_* λ_S_(*i*) + *β_1_* λ_S_^2^(*i*)), based on determined using permutation tests to account for partial non-independence of fitness contrasts (**Appendix S6**).

**Supplementary Figure 10.** Pairwise local-foreign fitness contrasts (*LF_i,j_*) as a function of demographic pair status of local (*i*) and foreign (*j*) populations grown at site *i.* For each cohort, the effect of demographic pair status (PS*_i,j_*) on pairwise fitness contrasts was analyzed in an ANOVA model: *LF_i,j_* = *β_0_ + β_1_* PS*_i,j_*. *P* values in each panel represent the significance of pair status based on permutation tests to account for partial non-independence of fitness contrasts (**Appendix S6**).

**Supplementary Figure 11.** Correlations between HA and LF metrics of local adaptation. **A:** Correlations between pairwise HA and LF fitness contrasts. **B:** Correlations between population-mean HA and LF local adaptation. In both panels, the dashed gray line represents a 1:1 between HA and LF metrics, and the solid black line is the best-fit linear regression relating HA and LF metrics.

**Supplementary Figure 12.** Relationship between pairwise fitness contrasts in Cohort 1 vs. Cohort 2 of the transplant experiment for **(A)** home-away contrasts and (**B**) local-foreign contrasts. The dashed gray line shows a 1:1 relationship between contrasts measured in the two years, and the solid black line shows the best-fit linear regression for observed data. Points below the dashed line represent source-site combinations that showed stronger signs of local adaptation in Cohort 1, points above the dashed line represent source-site combinations that showed stronger signs of local adaptation in Cohort 2, and points falling on the dashed line showed comparable strength of local adaptation in both transplant years.

**Supplementary Figure 13**. Linear regression of stochastic population growth rate (λ_s_) against deterministic population growth rate (λ) for the 10 populations used in the reciprocal transplant study (F_1,8_=49.2, *P* <0.0002).
