# Supplementary material for: Local Adaptation Is Highest in Populations With Stable Long‐Term Growth
Source: Ecol Lett. 2025 Feb 18;28(2):e70071. doi: 10.1111/ele.70071 (PMC11834371; doi:10.1111/ele.70071)
Supplement: Supplementary file 3 — Tables S1–S11 [file ELE-28-0-s001.docx]

## Supplementary tables

**Supplementary Table 1.** Axis loadings from the vital rate elasticity PCA (N=10) used in calculating demographic distance via method B (**Supplementary Figure 4**). Correlation coefficients indicate the relationship between each underlying vital rate elasticity and the first three principal component axes, which cumulatively explain >95% of variation in elasticity structures across populations. Vital rates are defined in **Appendix S1**. The vital rates with the highest absolute correlation coefficients with each PCA axis are indicated in bold.

| Vital rate | PCA axis  (cumulative % variance explained) | | |
| --- | --- | --- | --- |
|  | **PCA1**  **(67.5%)** | **PCA2**  **(92.4)** | **PCA3**  **(99.9)** |
| s1 | **-0.340** | 0.121 | -0.090 |
| s2 | -0.026 | **-0.565** | -0.206 |
| s3 | -0.026 | **-0.565** | -0.206 |
| s4 | 0.316 | 0.130 | **-0.407** |
| s5 | **0.342** | 0.0845 | 0.162 |
| s6 | 0.315 | 0.047 | **0.480** |
| g1 | -0.297 | 0.301 | -0.016 |
| g2 | -0.245 | **-0.402** | 0.160 |
| g3 | 0.268 | 0.157 | **-0.659** |
| sigma | **-0.340** | 0.121 | -0.090 |
| F | **-0.340** | 0.121 | -0.090 |
| phi | **-0.340** | 0.121 | -0.090 |

**Supplementary Table 2.** Axis loading from the environmental PCA (N=28) used in calculating environmental distance between focal populations. Correlation coefficients indicate the relationship between each underlying environmental variable and each of the first five principal component axes, which cumulatively explain >95% of the variance in environment across sites. For each environmental variable, mean and SD reflect the site mean and standard deviation in each site from 2005-2021 in temperature (T) or precipitation (P) measured in winter (W) or spring (S) seasons. RAD = solar radiation in Watts per m^2^. The environmental variables with the highest absolute correlation coefficients with each PCA axis are indicated in bold.

| Env. variable | PCA axis  (cumulative % variance explained) | | | | |
| --- | --- | --- | --- | --- | --- |
|  | **PCA1**  **(43.3%)** | **PCA2**  **(66.1%)** | **PCA3**  **(82.1%)** | **PCA4**  **(91.8%)** | **PCA5**  **(96.4%)** |
| Mean(T_S_) | **0.388** | 0.025 | **-0.431** | 0.153 | -0.072 |
| Mean(T_W_) | 0.299 | 0.210 | **-0.540** | 0.131 | -0.036 |
| Mean(P_S_) | -0.107 | **-0.397** | **-0.544** | 0.041 | 0.170 |
| Mean(P_W_) | **-0.358** | -0.278 | **-0.318** | 0.039 | -0.469 |
| SD(T_S_) | 0.156 | 0.073 | -0.182 | -0.920 | -0.120 |
| SD(T_W_) | 0.295 | **-0.462** | 0.178 | -0.248 | 0.003 |
| SD(P_S_) | -0.283 | **-0.388** | -0.108 | -0.097 | 0.667 |
| SD(P_W_) | **-0.419** | -0.193 | 0.019 | -0.042 | -0.488 |
| Winter solstice RAD | **-0.347** | **0.422** | -0.160 | -0.075 | 0.159 |
| Equinox RAD | **-0.364** | **0.367** | -0.150 | -0.179 | 0.148 |

**Supplementary Table 3.** The number of seeds planted in each source × site transplant combination.

|  |  | | | **Transplant location** | | | | | | | | | |
| --- | --- | --- | --- | --- | --- | --- | --- | --- | --- | --- | --- | --- | --- |
|  |  |  |  | **Set I** | | | | | **Set II** | | | | |
|  |  |  |  | **1**  **(BR)** | **2**  **(CF)** | **3**  **(CP3)** | **4**  **(DLW)** | **5**  **(S22)** | **1**  **(KYE)** | **2**  **(OKRE)** | **3**  **(OSR)** | **4**  **(FR)** | **5**  **(GCN)** |
| **Seed Source** | **Cohort 1**  **(N = 29,606)** | **Set**  **I** | **1 (BR)** | 769 | 768 | 768 | 544* | 768 | 792 | 0 | 0 | 0 | 0 |
|  |  |  | **2 (CF)** | 768 | 768 | 767 | 544* | 767 | 0 | 791 | 0 | 0 | 0 |
|  |  |  | **3 (CP3)** | 768 | 768 | 767 | 544* | 767 | 0 | 0 | 792 | 0 | 0 |
|  |  |  | **4 (DLW)** | 768 | 768 | 768 | 544* | 768 | 0 | 0 | 0 | 792 | 0 |
|  |  |  | **5 (S22)** | 768 | 768 | 767 | 544* | 768 | 0 | 0 | 0 | 0 | 792 |
|  |  | **Set II** | **1 (KYE)** | 768 | 0 | 0 | 0 | 0 | 786 | 0 | 0 | 0 | 0 |
|  |  |  | **2 (OKRE)** | 0 | 768 | 0 | 0 | 0 | 0 | 792 | 0 | 0 | 0 |
|  |  |  | **3 (OSR)** | 0 | 0 | 744 | 0 | 0 | 0 | 0 | 792 | 0 | 0 |
|  |  |  | **4 (FR)** | 0 | 0 | 24 | 544* | 0 | 0 | 0 | 0 | 792 | 0 |
|  |  |  | **5 (GCN)** | 0 | 0 | 0 | 0 | 768 | 0 | 0 | 0 | 0 | 792 |
|  | **Cohort 2**  **(N = 30,386)** | **Set**  **I** | **1 (BR)** | 766 | 768 | 768 | 685* | 758 | 790 | 0 | 0 | 0 | 0 |
|  |  |  | **2 (CF)** | 767 | 768 | 768 | 687* | 758 | 0 | 792 | 0 | 0 | 0 |
|  |  |  | **3 (CP3)** | 767 | 768 | 767 | 685* | 757 | 0 | 0 | 791 | 0 | 0 |
|  |  |  | **4 (DLW)** | 767 | 768 | 768 | 688* | 760 | 0 | 0 | 0 | 786 | 0 |
|  |  |  | **5 (S22)** | 767 | 768 | 767 | 688* | 758 | 0 | 0 | 0 | 0 | 792 |
|  |  | **Set II** | **1 (KYE)** | 765 | 0 | 0 | 0 | 0 | 792 | 0 | 0 | 0 | 0 |
|  |  |  | **2 (OKRE)** | 0 | 768 | 0 | 0 | 0 | 0 | 791 | 0 | 0 | 0 |
|  |  |  | **3 (OSR)** | 0 | 0 | 768 | 0 | 0 | 0 | 0 | 792 | 0 | 0 |
|  |  |  | **4 (FR)** | 0 | 0 | 0 | 686* | 0 | 0 | 0 | 0 | 788 | 0 |
|  |  |  | **5 (GCN)** | 0 | 0 | 0 | 0 | 757 | 0 | 0 | 0 | 0 | 792 |

*Cattle trampling destroyed some experimental infrastructure at one site from Set I (Delonegha West, i.e. DLW), reducing replication from 96 to 68 blocks in Cohort 1, and 96 to 86 blocks in Cohort 2.

**Supplementary Table 4.** Summary of linear models linking fruit mass to seed number in the second year of the transplant experiment. In predictive models, coefficient estimates were used to the maximum level of precision provided by the output of these linear models (7-8 decimal places); for concision, estimates shown here are rounded to 3 decimal places. Site = transplant site; pop = source population; dam = herbivore damage to fruit (u=undamaged, d=damaged); SE = standard error.

| **Site** | **Pop** | **Dam** | **Intercept (SE)** | **β_Fruit_weight_ (SE)** | **R^2^** | ***P*** | **n** |
| --- | --- | --- | --- | --- | --- | --- | --- |
| BR | BR | u | -7.247 (1.999) | 1.546 (0.067) | 0.962 | 1.93×10^-16^ | 23 |
| BR | BR | d | -3.649 (2.806) | 0.421 (0.125) | 0.506 | 0.006 | 13 |
| BR | CF | u | -7.355 (3.468) | 1.522 (0.061) | 0.967 | 4.62×10^-17^ | 23 |
| BR | CF | d | 0.176 (1.438) | 0.130 (0.051) | 0.331 | 0.025 | 15 |
| BR | CP3 | u | -6.106 (4.513) | 1.580 (0.160) | 0.867 | 5.76×10^-8^ | 17 |
| BR | CP3 | d | -1.121 (2.264) | 0.298 (0.193) | 0.146 | 0.144 | 16 |
| BR | DLW | u | -11.073 (5.956) | 1.567 (0.066) | 0.968 | 1.31×10^-15^ | 21 |
| BR | DLW | d | -3.765 (3.206) | 0.881 (0.167) | 0.622 | 6.06×10^-5^ | 19 |
| BR | S22 | u | 15.734 (9.096) | 0.688 (0.212) | 0.306 | 0.003 | 26 |
| BR | S22 | d | -2.699 (1.125) | 0.533 (0.074) | 0.867 | 9.11×10^-5^ | 10 |
| BR | KYE | u | -7.632 (4.581) | 1.607 (0.101) | 0.924 | 3.29×10^-13^ | 23 |
| BR | KYE | d | 0.026 (4.782) | 0.485 (0.136) | 0.460 | 0.003 | 17 |
| CF | BR | u | -6.678 (8.594) | 0.956 (0.147) | 0.669 | 1.86×10^-6^ | 23 |
| CF | BR | d | 11.737 (9.705) | 0.380 (0.153) | 0.359 | 0.030 | 13 |
| CF | CF | u | -9.725 (3.989) | 1.125 (0.052) | 0.961 | 6.72×10^-15^ | 21 |
| CF | CF | d | -10.373 (6.401) | 1.058 (0.127) | 0.832 | 8.69×10^-7^ | 16 |
| CF | CP3 | u | 1.390 (5.366) | 1.043 (0.095) | 0.903 | 5.90×10^-8^ | 15 |
| CF | CP3 | d | -0.226 (2.981) | 0.480 (0.057) | 0.826 | 4.52×10^-7^ | 17 |
| CF | DLW | u | -6.423 (4.918) | 1.032 (0.058) | 0.941 | 9.87×10^-14^ | 22 |
| CF | DLW | d | -8.134 (2.790) | 0.909 (0.060) | 0.9387 | 1.83×10^-10^ | 17 |
| CF | S22 | u | 3.472 (4.676) | 0.644 (0.090) | 0.750 | 1.67×10^-6^ | 19 |
| CF | S22 | d | -7.103 (2.492) | 0.930 (0.106) | 0.874 | 2.76×10^-6^ | 13 |
| CF | OKRE | u | 0.549 (6.436) | 0.978 (0.072) | 0.897 | 7.75×10^-12^ | 23 |
| CF | OKRE | d | -5.442 (10.165) | 0.900 (0.137) | 0.828 | 0.0001 | 11 |
| CP3 | BR | u | -13.502 (7.231) | 1.025 (0.045) | 0.946 | 6.14×10^-20^ | 31 |
| CP3 | BR | d | -4.394 (4.661) | 0.484 (0.155) | 0.495 | 0.011 | 12 |
| CP3 | CF | u | -11.093 (11.052) | 1.192 (0.116) | 0.784 | 3.80×10^-11^ | 31 |
| CP3 | CF | d | 0 (NA) | 0 (NA) | NA | NA | 2 |
| CP3 | CP3 | u | -4.391 (7.855) | 1.321 (0.068) | 0.926 | 1.58×10^-18^ | 32 |
| CP3 | CP3 | d | -1.541 (3.788) | 0.430 (0.198) | 0.486 | 0.082 | 7 |
| CP3 | DLW | u | -12.513 (6.775) | 1.444 (0.048) | 0.967 | 7.48×10^-24^ | 32 |
| CP3 | DLW | d | 0.587 (2.516) | 0.201 (0.150) | 0.264 | 0.238 | 7 |
| CP3 | S22 | u | -7.498 (8.160) | 1.507 (0.085) | 0.918 | 9.04×10^-17^ | 30 |
| CP3 | S22 | d | 1.277 (4.076) | 0.362 (0.228) | 0.386 | 0.188 | 6 |
| CP3 | OSR | u | -18.819 (15.649) | 1.430 (0.074) | 0.917 | 6.00×10^-20^ | 36 |
| CP3 | OSR | d | 1.502 (2.010) | 0.371 (0.084) | 0.661 | 0.001 | 12 |
| DLW | BR | u | 7.510 (16.028) | 0.287 (0.575) | 0.077 | 0.652 | 5 |
| DLW | BR | d | -6.945 (3.744) | 0.777 (0.150) | 0.817 | 0.002 | 8 |
| DLW | CF | u | -2.898 (7.630) | 0.856 (0.137) | 0.673 | 5.28×10^-6^ | 21 |
| DLW | CF | d | 0.511 (1.904) | 0.251 (0.065) | 0.500 | 0.001 | 17 |
| DLW | CP3 | u | -6.284 (8.099) | 1.236 (0.235) | 0.716 | 0.0003 | 13 |
| DLW | CP3 | d | -6.206 (3.904) | 1.144 (0.239) | 0.766 | 0.002 | 9 |
| DLW | DLW | u | -0.879 (13.799) | 1.172 (0.130) | 0.871 | 1.12×10^-6^ | 14 |
| DLW | DLW | d | -3.185 (5.237) | 0.947 (0.159) | 0.733 | 4.68×10^-5^ | 15 |
| DLW | S22 | u | -3.477 (18.851) | 1.508 (0.358) | 0.816 | 0.014 | 6 |
| DLW | S22 | d | -0.755 (11.845) | 0.836 (0.459) | 0.453 | 0.143 | 6 |
| DLW | FR | u | -1.047 (4.654) | 1.154 (0.046) | 0.967 | 4.49×10^-17^ | 23 |
| DLW | FR | d | -1.509 (2.382) | 0.798 (0.073) | 0.902 | 6.24×10^-8^ | 15 |
| S22 | BR | u | 13.962 (4.807) | 0.612 (0.048) | 0.855 | 2.88×10^-13^ | 30 |
| S22 | BR | d | 0.204 (3.454) | 0.677 (0.235) | 0.625 | 0.034 | 7 |
| S22 | CF | u | -4.663 (2.771) | 1.051 (0.025) | 0.985 | 3.91×10^-26^ | 29 |
| S22 | CF | d | -1.084 (2.064) | 0.645 (0.131) | 0.752 | 0.001 | 10 |
| S22 | CP3 | u | -6.874 (8.221) | 1.354 (0.073) | 0.920 | 5.60×10^-18^ | 32 |
| S22 | CP3 | d | -0.846 (2.910) | 0.758 (0.142) | 0.759 | 0.0005 | 11 |
| S22 | DLW | u | -20.741 (9.357) | 1.480 (0.047) | 0.967 | 4.69×10^-26^ | 35 |
| S22 | DLW | d | -5.907 (5.592) | 1.560 (0.385) | 0.646 | 0.003 | 11 |
| S22 | S22 | u | -16.210 (3.556) | 1.383 (0.013) | 0.997 | 2.01×10^-40^ | 32 |
| S22 | S22 | d | -5.635 (4.873) | 1.016 (0.325) | 0.494 | 0.011 | 12 |
| S22 | GCN | u | -2.005 (3.840) | 1.114 (0.027) | 0.978 | 1.91×10^-32^ | 39 |
| S22 | GCN | d | -5.716 (1.043) | 1.112 (0.048) | 0.975 | 1.33×10^-12^ | 16 |
| FR | FR | u | -8.785 (4.705) | 1.539 (0.058) | 0.974 | 1.52×10^-16^ | 21 |
| FR | FR | d | -0.438 (5.809) | 0.619 (0.252) | 0.334 | 0.031 | 14 |
| FR | DLW | u | 2.666 (5.365) | 0.889 (0.118) | 0.740 | 2.80×10^-7^ | 22 |
| FR | DLW | d | -6.048 (2.140) | 0.906 (0.109) | 0.803 | 2.15×10^-7^ | 19 |
| GCN | GCN | u | -7.258 (6.231) | 1.481 (0.077) | 0.929 | 1.17×10^-17^ | 30 |
| GCN | GCN | d | -5.218 (3.617) | 0.892 (0.085) | 0.956 | 0.0001 | 7 |
| GCN | S22 | u | -15.960 (4.968) | 1.859 (0.056) | 0.978 | 3.59×10^-22^ | 27 |
| GCN | S22 | d | -4.452 (11.604) | 1.276 (0.711) | 0.446 | 0.147 | 6 |
| KYE | KYE | u | 5.264 (4.571) | 1.005 (0.071) | 0.931 | 4.29×10^-10^ | 17 |
| KYE | KYE | d | -6.974 (3.892) | 0.947 (0.158) | 0.720 | 3.27×10^-5^ | 16 |
| KYE | BR | u | -12.726 (5.822) | 1.502 (0.134) | 0.920 | 2.32×10^-7^ | 13 |
| KYE | BR | d | -3.238 (4.506) | 0.739 (0.129) | 0.660 | 2.40×10^-5^ | 19 |
| OKRE | OKRE | u | -0.889 (7.432) | 0.867 (0.285) | 0.698 | 0.038 | 6 |
| OKRE | OKRE | d | 0.265 (0.275) | -0.002 (0.006) | 0.054 | 0.707 | 5 |
| OKRE | CF | u | -3.994 (3.154) | 0.462 (0.089) | 0.770 | 0.0008 | 10 |
| OKRE | CF | d | 0.2025 (1.443) | 0.057 (0.108) | 0.084 | 0.637 | 5 |
| OSR | OSR | u | 1.337 (9.614) | 1.409 (0.051) | 0.965 | 6.60×10^-22^ | 30 |
| OSR | OSR | d | 9.949 (NA) | 0.506 (NA) | 1 | NA | 2 |
| OSR | CP3 | u | 0.228 (14.133) | 1.349 (0.085) | 0.904 | 2.97×10^-15^ | 29 |
| OSR | CP3 | d | -27.557 (3.491) | 1.717 (0.072) | 0.997 | 0.002 | 4 |

**Supplementary Table 5.** Outcomes of analyses of deviance for *aster* models of absolute fitness in the reciprocal transplant experiments. Within each cohort and site combination, nested models with (nested model 1) and without (nested model 2) effects of source population were compared to test the significance of source population. For each individual model, the goodness-of-fit is estimated as -2log(likelihood). The test statistic for model comparison is the deviance of the goodness-of-fit between the two models.

| **Cohort** | **Site**  **(N)** | **Individual models** | | | **Model comparison** | | | |
| --- | --- | --- | --- | --- | --- | --- | --- | --- |
|  |  | **Nested model** | **df** | **-2log(lik)** | **df** | **Deviance** | ***P*** | |
| 1 | BR | 1 | 6 | 787187 |  |  |  |  |
|  | (4,475) | 2 | 11 | 787200 | 5 | 13.19 | 0.02167 | * |
|  | CF | 1 | 6 | 635486 |  |  |  |  |
|  | (4,215) | 2 | 11 | 635672 | 5 | 185.91 | < 2.2×10^-16^ | *** |
|  | C3 | 1 | 6 | 116643 |  |  |  |  |
|  | (4,570) | 2 | 11 | 116706 | 5 | 63.185 | 2.67×10^-12^ | *** |
|  | DLW | 1 | 6 | 1166246 |  |  |  |  |
|  | (3,243) | 2 | 11 | 1166468 | 5 | 221.4 | < 2.2×10^-16^ | *** |
|  | S22 | 1 | 6 | 219931 |  |  |  |  |
|  | (4,581) | 2 | 11 | 219948 | 5 | 16.14 | 0.006455 | ** |
|  | KYE | 1 | 6 | 38069 |  |  |  |  |
|  | (1,562) | 2 | 7 | 38074 | 1 | 5.2454 | 0.022 | * |
|  | OKRE | 1 | 6 | 50850 |  |  |  |  |
|  | (1,521) | 2 | 7 | 50858 | 1 | 8.1202 | 0.004377 | ** |
|  | OSR | 1 | 6 | 30329 |  |  |  |  |
|  | (1,535) | 2 | 7 | 30329 | 1 | 0.056596 | 0.812 |  |
|  | FR | 1 | 6 | 192407 |  |  |  |  |
|  | (1,515) | 2 | 7 | 192409 | 1 | 2.268 | 0.1321 |  |
|  | GCN | 1 | 6 | 97596 |  |  |  |  |
|  | (1,576) | 2 | 7 | 97649 | 1 | 53.023 | 3.30×10^-13^ | *** |
| 2 | BR | 1 | 5 | 44057 |  |  |  |  |
|  | (4,450) | 2 | 10 | 44216 | 5 | 159.85 | < 2.2×10^-16^ | *** |
|  | CF | 1 | 5 | 81447 |  |  |  |  |
|  | (4,465) | 2 | 10 | 81632 | 5 | 184.51 | < 2.2×10^-16^ | *** |
|  | C3 | 1 | 5 | 280047 |  |  |  |  |
|  | (4,564) | 2 | 10 | 280135 | 5 | 88.64 | < 2.2×10^-16^ | *** |
|  | DLW | 1 | 5 | 18956 |  |  |  |  |
|  | (4,055) | 2 | 10 | 19007 | 5 | 51.064 | 8.39×10^-10^ | *** |
|  | S22 | 1 | 5 | 251926 |  |  |  |  |
|  | (4,521) | 2 | 10 | 252044 | 5 | 117.89 | < 2.2×10^-16^ | *** |
|  | KYE | 1 | 5 | 5106 |  |  |  |  |
|  | (1,573) | 2 | 6 | 5107.3 | 1 | 1.2273 | 0.2679 |  |
|  | OKRE | 1 | 5 | -1565.4 |  |  |  |  |
|  | (1,581) | 2 | 6 | -1565.4 | 1 | 0.00060116 | 0.9804 |  |
|  | OSR | 1 | 5 | 176477 |  |  |  |  |
|  | (1,576) | 2 | 6 | 176488 | 1 | 10.462 | 0.001218 | ** |
|  | FR | 1 | 5 | 23382 |  |  |  |  |
|  | (1,535) | 2 | 6 | 23389 | 1 | 6.8044 | 0.009094 | ** |
|  | GCN | 1 | 4 | 133779 |  |  |  |  |
|  | (1,567) | 2 | 5 | 133803 | 1 | 23.567 | 1.21×10^-6^ | *** |

**Supplementary Table 6.** Empirical and permuted statistics testing for variation in population-mean HA local adaptation in response to demography in Cohorts 1 and 2 of the reciprocal transplant experiment. β values are partial regression coefficients. Bold red text shows significant effects, and italic red text shows marginally significant effects. These models employ weighted regressions accounting for differences in the amount of information used to estimate population-mean local adaptation in Set I vs. Set II populations (weights: Set I populations = 5; Set II = populations 1).

| Pred | Cohort 1 | | | | | Cohort 2 | | | | |
| --- | --- | --- | --- | --- | --- | --- | --- | --- | --- | --- |
|  | **Empirical model**  **(N_obs_=10)** | | | **Permutation test**  **(N_perm_=5000)** | | **Empirical model**  **(N_obs_=10)** | | | **Permutation test**  **(N_perm_=5000)** | |
|  | **β**  **(±SE)** | ***F* (df)** | ***P*** | **Thresh. *F*** | **Perm. *P*** | **β**  **(±SE)** | ***F***  **(df)** | ***P*** | **Thresh. *F*** | **Perm. *P*** |
| λ_S_ | 12.536 (5.582) | 5.0  (1,7) | *0.0596* | 4.70 | **0.0450** | 8.651 (3.332) | 6.7 (1,7) | **0.0356** | 5.12 | **0.0274** |
| λ_S_^2^ | -5.819 (2.573) | 5.1  (1,7) | *0.0582* | 4.39 | **0.0386** | -4.213 (1.536) | 7.5 (1,7) | **0.0288** | 4.67 | **0.0172** |

**Supplementary Table 7.** Empirical and permuted statistics testing for variation in population-mean LF local adaptation in response to demography in Cohorts 1 and 2 of the reciprocal transplant experiment. β values are partial regression coefficients. These models employ weighted regressions accounting for differences in the amount of information used to estimate population-mean local adaptation in Set I vs. Set II populations (weights: Set I populations = 5; Set II populations = 1).

| Pred | Cohort 1 | | | | | Cohort 2 | | | | |
| --- | --- | --- | --- | --- | --- | --- | --- | --- | --- | --- |
|  | **Empirical model**  **(N_obs_=10)** | | | **Permutation test**  **(N_perm_=5000)** | | **Empirical model**  **(N_obs_=10)** | | | **Permutation test**  **(N_perm_=5000)** | |
|  | **β**  **(±SE)** | ***F* (df)** | ***P*** | **Thresh. *F*** | **Perm. *P*** | **β**  **(±SE)** | ***F* (df)** | ***P*** | **Thresh. *F*** | **Perm. *P*** |
| λ_S_ | 10.149 (8.277) | 1.5  (1,7) | 0.2597 | 4.84 | 0.2308 | -10.266 (10.790) | 0.9 (1,7) | 0.3731 | 4.90 | 0.3404 |
| λ_S_^2^ | -4.553 (3.815) | 1.4  (1,7) | 0.2716 | 4.52 | 0.2250 | 3.579 (4.973) | 0.5 (1,7) | 0.4950 | 4.56 | 0.4438 |

**Supplementary Table 8.** Empirical and permuted statistics testing differences in HA (**A**) and LF (**B**) pairwise fitness contrasts across demographically paired vs. unpaired source-site combinations in Cohorts 1 and 2 of the reciprocal transplant experiment. Bold red text shows significant effects and italic red text shows marginally significant effects.

| Model type | Predictor | Cohort 1 | | | | Cohort 2 | | | |
| --- | --- | --- | --- | --- | --- | --- | --- | --- | --- |
|  |  | **Empirical model (N_obs_=30)** | | **Permutation  test**  **(N_perm_=5000)** | | **Empirical model**  **(N_obs_=30)** | | **Permutation  test**  **(N_perm_=5000)** | |
|  |  | ***F***  **(df)** | ***P*** | **Thresh. *F*** | **Perm. *P*** | ***F***  **(df)** | ***P*** | **Thresh. *F*** | **Perm. *P*** |
| A) HA | Categorical demographic pair status | 5.01  (1,28) | **0.0333** | 4.50 | **0.0374** | 6.03 (1,28) | **0.0206** | 4.84 | **0.0308** |
| B) LF | Categorical demographic pair status | 3.88  (1,28) | *0.0590* | 4.43 | *0.0686* | 1.48  (1,28) | 0.2337 | 4.43 | 0.2498 |

**Supplementary Table 9.** Empirical and permuted statistics testing for variation in HA pairwise fitness contrasts in response to geographic, environmental, and demographic distance in Cohorts 1 and 2 of the reciprocal transplant experiment. In these models, demographic distance is estimated as pairwise distance in vital rate elasticity space (**Figure 1D**). β values are partial regression coefficients linking HA contrasts to each putative driver after accounting for potential covariance among drivers. Bold red text shows significant effects and italic red text shows marginally significant effects.

| Predictor | Cohort 1 | | | | | Cohort 2 | | | | |
| --- | --- | --- | --- | --- | --- | --- | --- | --- | --- | --- |
|  | **Empirical model**  **(N_obs_=30)** | | | **Permutation test**  **(N_perm_=5000)** | | **Empirical model**  **(N_obs_=30)** | | | **Permutation test**  **(N_perm_=5000)** | |
|  | **β**  **(±SE)** | ***F***  **(df)** | ***P*** | **Thresh. *F*** | **Perm. *P*** | **β**  **(±SE)** | ***F***  **(df)** | ***P*** | **Thresh. *F*** | **Perm. *P*** |
| Geodist | 0.142 (0.107) | 1.74 (1,26) | 0.1985 | 4.09 | 0.1884 | 0.180 (0.076) | 5.53  (1,26) | **0.0266** | 3.92 | **0.0228** |
| EnvDist | 0.207 (0.104) | 3.93  (1,26) | *0.0580* | 4.08 | *0.0548* | 0.020 (0.074) | 0.07  (1,26) | 0.7907 | 4.14 | 0.7996 |
| DemDistB  (VR elas. PCA) | 0.056 (0.105) | 0.28  (1,26) | 0.6007 | 5.18 | 0.6334 | 0.040 (0.075) | 0.29  (1,26) | 0.5958 | 5.06 | 0.6380 |

**Supplementary Table 10.** Empirical and permuted statistics testing for variation in LF pairwise fitness contrasts in response to geographic, environmental, and demographic distance in Cohorts 1 and 2 of the reciprocal transplant experiment. In this model, demographic distance is estimated as pairwise distance in vital rate elasticity space (**Figure 1D**). β values are partial regression coefficients linking LF contrasts to each putative driver after accounting for potential covariance among drivers.

| Predictor | Cohort 1 | | | | | Cohort 2 | | | | |
| --- | --- | --- | --- | --- | --- | --- | --- | --- | --- | --- |
|  | **Empirical model**  **(N_obs_=30)** | | | **Permutation test**  **(N_perm_=5000)** | | **Empirical model**  **(N_obs_=30)** | | | **Permutation test**  **(N_perm_=5000)** | |
|  | **β**  **(±SE)** | ***F***  **(df)** | ***P*** | **Thresh. *F*** | **Perm.**  ***P*** | **β**  **(±SE)** | ***F***  **(df)** | ***P*** | **Thresh. *F*** | **Perm. *P*** |
| Geodist | 0.121 (0.135) | 0.80  (1,26) | 0.3783 | 3.93 | 0.3778 | 0.090 (0.119) | 0.57  (1,26) | 0.4573 | 3.99 | 0.4573 |
| EnvDist | 0.180 (0.131) | 1.87  (1,26) | 0.1835 | 4.01 | 0.1870 | -0.008 (0.116) | 0.01  (1,26) | 0.9434 | 4.28 | 0.9434 |
| DemDistB  (VR elas. PCA) | 0.072 (0.132) | 0.30  (1,26) | 0.5914 | 4.82 | 0.6260 | 0.072 (0.116) | 0.38  (1,26) | 0.5440 | 5.03 | 0.5440 |

**Supplementary Table 11.** Germination success in the first year following sowing during the reciprocal transplant experiment.

| **Transplant site** | | **Germination (%)** | |
| --- | --- | --- | --- |
|  |  | **Cohort 1** | **Cohort 2** |
| **Set**  **I** | **BR** | 27.8 | 43.4 |
|  | **CF** | 37.5 | 45.3 |
|  | **CP3** | 18.2 | 45.3 |
|  | **DLW** | 26.5 | 27.9 |
|  | **S22** | 25.3 | 42.8 |
| **Set II** | **KYE** | 33.5 | 39.1 |
|  | **OKRE** | 33.0 | 24.0 |
|  | **OSR** | 6.4 | 40.2 |
|  | **FR** | 28.3 | 40.5 |
|  | **GCN** | 28.7 | 47.5 |
